# An integrative pan‐cancer analysis of the molecular and biological features of glycosyltransferases

**DOI:** 10.1002/ctm2.872

**Published:** 2022-07-08

**Authors:** Yin Li, Youpei Lin, Ling Aye, Liangqing Dong, Chenhao Zhang, Fanghua Chen, Yinkun Liu, Jia Fan, Qiang Gao, Haojie Lu, Chunlai Lu, Shu Zhang

**Affiliations:** ^1^ Department of Thoracic Surgery Zhongshan Hospital Fudan University Shanghai P. R. China; ^2^ Liver Cancer Institute, Zhongshan Hospital Key Laboratory of Carcinogenesis and Cancer Invasion (Ministry of Education) Fudan University Shanghai P. R. China; ^3^ Institutes of Biomedical Sciences Fudan University Shanghai P. R. China; ^4^ NHC Key Laboratory of Glycoconjugates Research and Department of Chemistry Fudan University Shanghai P. R. China

Dear Editor,

Glycosyltransferases (GTs) played important roles in cancer development and progression.^1^, ^2^ Here, we conducted a pan‐cancer analysis of GTs (Supporting information Table S1)^3^ based on the TCGA data, CCLE data, single‐cell RNA sequencing datasets and our proteogenomic resource, aiming to characterize the molecular features, biological functions and clinical implications of GTs across cancer types.

The overall mutation frequency of GTs was relatively low (0.0–11.6%). Cancer types with higher global mutation burdens exhibited higher mutation frequencies of GTs. The highest mutation frequencies were observed in UCEC (ALG13, 11.6%), SKCM (FUT9, 10.6%) and SKCM (GALNT13, 10.6%) (Figure 1A, Supporting information Figure S1A and Table S2). Survival analysis revealed that the UGGT2 mutation in COAD was linked to worse clinical outcomes, while the ALG13 mutation in UCEC was associated with better survival (Figure 1B). Furthermore, COAD patients with UGGT2 mutation showed enrichment of chronic inflammatory response, while UCEC patients with ALG13 mutation showed downregulation of response to cAMP (Figure 1C). Analysis of CCLE drug sensitivity showed colon cancer cell lines with UGGT2 mutation were resistant to EGFR inhibitors (Erlotinib and Lapatinib), and endometrial cancer cell lines with ALG13 mutation were sensitive to Panobinostat and Sorafenib (Figure 1D and Supporting information Figure S1B). It was worth noting that ALG1/2/11/14 were essential in cell survival across various cancer cell lines (Figure 1E). Widespread copy‐number variations (CNVs) of GTs were found across cancer types (Figure 1F, Supporting information Figure S2 and Table S3). In addition, mutation status and CNVs of GTs in cancer cell lines of CCLE displayed a similar pattern to the TCGA pan‐cancer cohort (Supporting information Figure S3).

**FIGURE 1:**
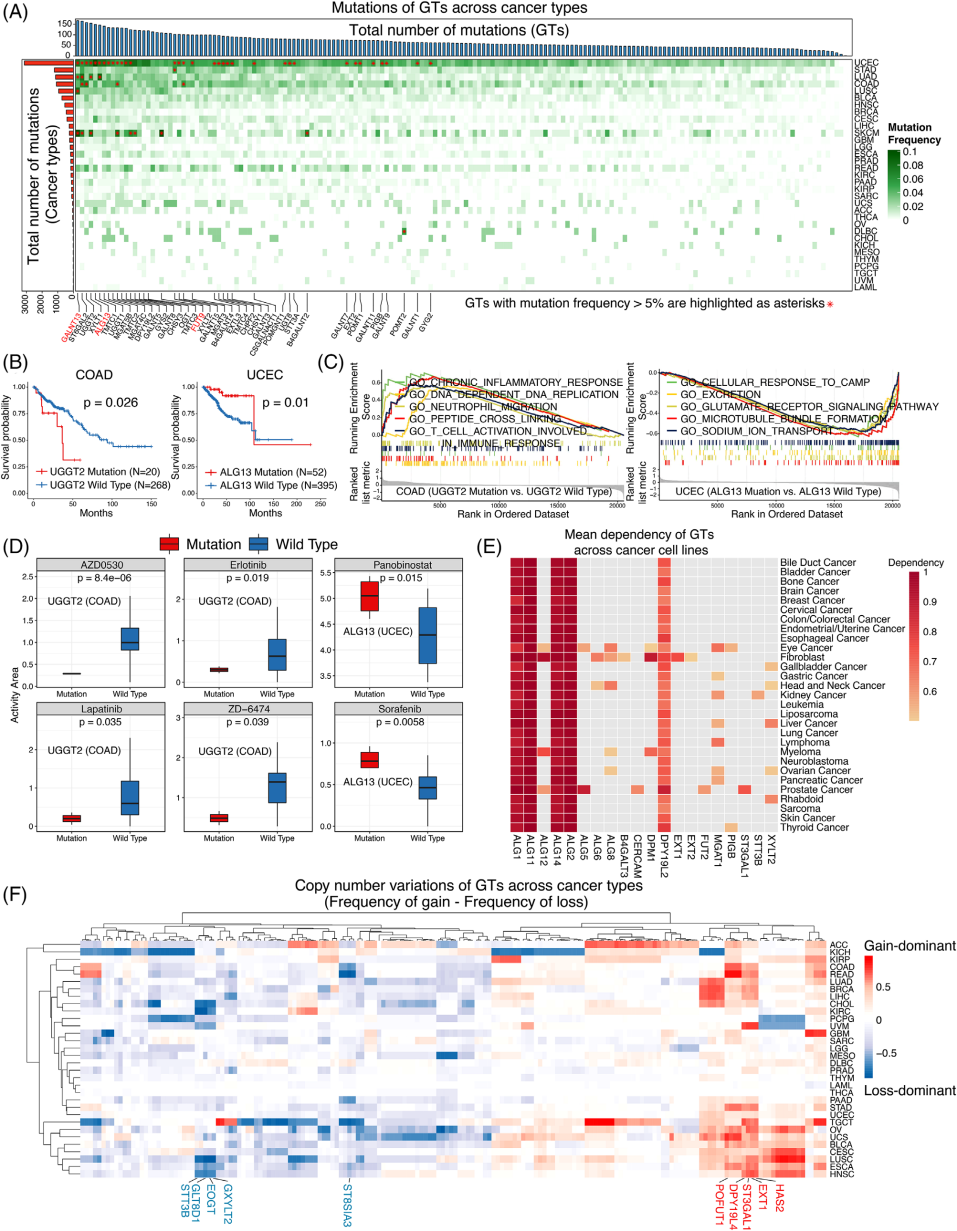
Genetic features of GTs across the pan‐cancer cohort. (A) Mutations of GTs across 33 cancer types. GTs with mutation frequency >5% are highlighted as asterisks, and a total of 41 GTs had more than 5.0% of mutation frequencies across the pan‐cancer cohort. The horizontal bar on the top indicates the total number of mutations of each GT across cancer types. The vertical bar on the left indicates the total number of mutations of all GTs in each cancer. The top three mutated GTs are highlighted in red. Cancer types with higher global mutation burdens, such as UCEC, COAD and STAD, exhibited higher mutation frequencies of GTs. (B) Survival analysis of patients with or without UGGT2/ALG13 mutations in COAD/UCEC. (C) GSEA (GO biological process) analysis of patients with UGGT2/ALG13 mutation in COAD/UCEC (adjusted *p* < 0.05). COAD patients with UGGT2 mutation showed enrichment of chronic inflammatory response, DNA replication, and neutrophil migration, while UCEC patients with ALG13 mutation showed downregulation of response to cAMP, glutamate signaling, and microtubule bundle formation. (D) Drug sensitivity comparison between mutation and wild type groups of UGGT2/ALG13 in COAD/UCEC cell lines. Drug sensitivity is measured using an activity area, with a higher number indicating higher sensitivity. (E) Essentiality of GTs in cell survival. (F) The CNVs landscape of GTs across cancer types. The differences between the CNV gain frequency and the CNV loss frequency of each GT in each cancer were calculated, and a GT CNV‐gain‐dominant as the difference value should be greater than 0.5 or CNV‐loss‐dominant as the difference value less than −0.5. DPY19L4, ST3GAL1, EXT1, HAS2, and POFUT1 exhibited widespread CNV gains in nearly half of the cancer types. In contrast, ST8SIA3, STT3B, EOGT, GXYLT2, and GLT8D1 displayed prevalent CNV losses

Widespread gene expression changes of GTs in tumors were observed (Figure 2A, Supporting information Figures S4, S5A and Table S4), among them, three GTs displayed consistent expression alterations in 16 cancer types, including upregulation of ALG3, and downregulation of B3GALT2 and ST6GALNAC3 (Figure 2B). Functional analyses of these three GTs showed a strong similarity in biological functions across cancer types (Figure 2C). In addition, the expression of GTs was tightly associated with patients' prognosis (Figure 2A, Supporting information Figure S5B and Table S5). For example, decreased expression of GYS2 conveyed poor prognosis in LIHC (Figure 2D), which was consistent with previous findings that GYS2 could inhibit tumor growth via a negative feedback loop with p53.^4^ In LUAD, B3GNT3 and GALNT14 were aberrantly expressed and associated with overall survival in different LUAD cohorts (Supporting information Figure S6). The prognostic significance of GTs was also evaluated in two external cohorts of patients receiving immune checkpoint inhibitors,^5^, ^6^ and higher expression of B3GNT4 indicated worse clinical outcomes in both cohorts (Supporting information Figure S7).

**FIGURE 2:**
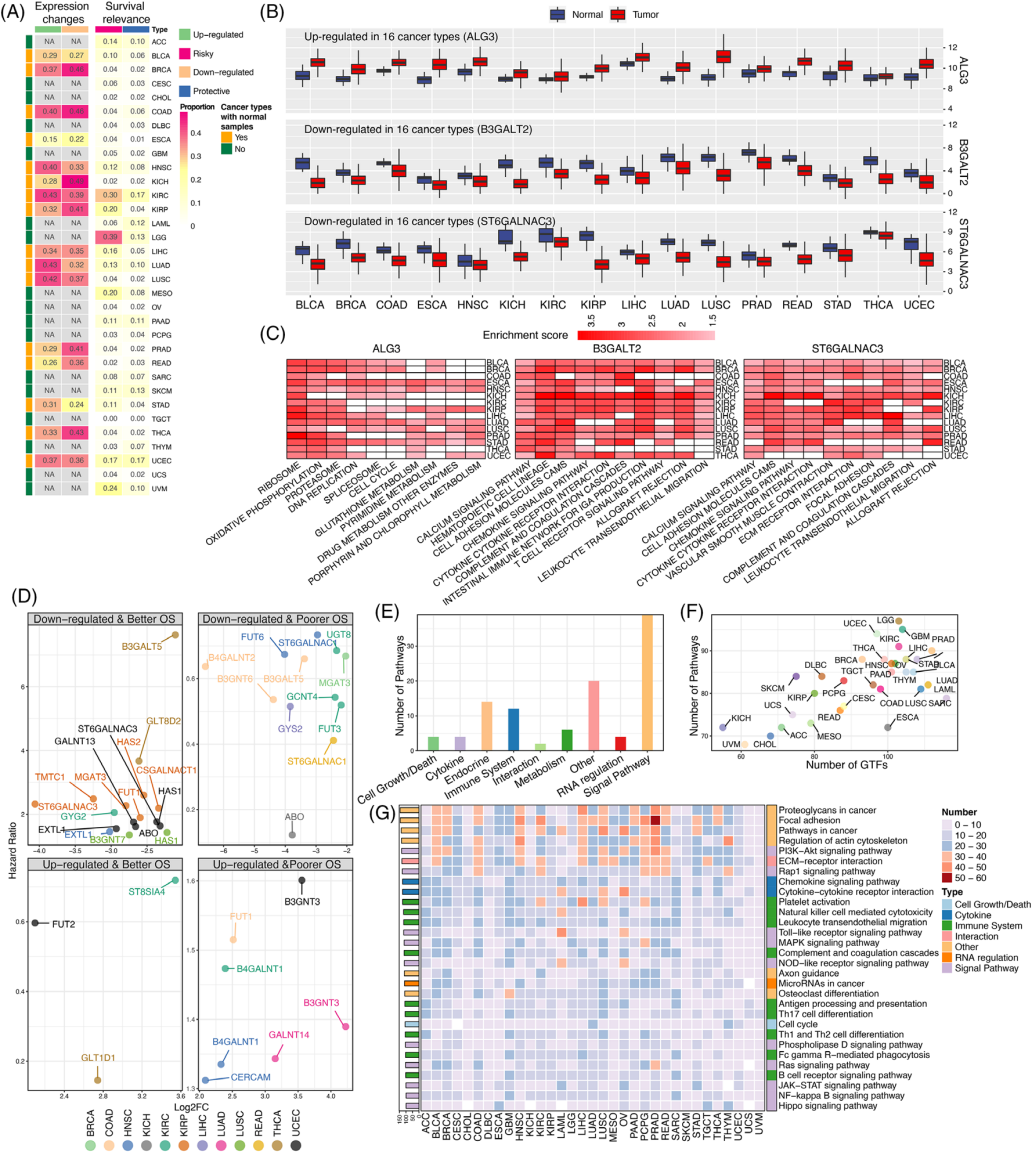
Expression features of GTs and GT‐pathway interaction landscape. (A) The proportion of GTs that were aberrantly expressed (left panel, adjusted *p* < 0.05), as well as the proportion of GTs that were significantly associated with patients’ prognosis in different cancer types (right panel, risky GTs were defined as the hazard ratios greater than 1, while protective GTs were defined as the hazard ratios lesser than 1). In total, 39% of GTs were identified as risk factors in LGG, followed by KIRC, with 30% of GTs being associated with worse overall survival (OS). KIRC and UCEC ranked the top in terms of the proportions of GTs that were identified as protective factors. (B) Three GTs (ALG3, B3GALT2, and ST6GALNAC3) showed consistent expression alterations across 16 cancers (adjusted *p* < 0.05). (C) GSEA analysis of ALG3, B3GALT2, and ST6GALNAC3 (adjusted *p* < 0.05) in 16 cancer types. The red color represents that the GT is significantly associated with this pathway in a given cancer type, and deeper red (higher enrichment score) indicates stronger associations. The white color indicates that this GT is not associated with the pathway in this cancer type. Specifically, the expression change of ALG13 was associated with ribosome in 15 cancer types. B3GALT2 was related to cell interaction and immune‐related pathways. ST6GALNAC3 was linked with calcium pathway and cell adhesion. (D) GTs with both changed expression (|Log_2_ fold‐change| > 2) and OS relevance. (E) Distribution of GT‐relevant pathways across different pathway categories. (F) The number of GTs versus the number of pathways in different cancer types. Stronger impacts of GTs on cancer‐relevant pathways occurred in LGG, GBM, UCEC, KIRC, PRAD, and LIHC. (G) Top 30 GTs‐related biological pathways across cancer types. The right column annotates the pathways categories. The left bar represents the total number of GTs involved in each pathway across different cancer types. Immune‐related pathways, such as chemokine signaling pathway, cytokine–cytokine receptor interaction, leukocyte transendothelial migration, antigen processing and presentation, as well as PD‐L1 expression and PD‐1 checkpoint pathway, were associated with GTs in various cancer types

The pan‐cancer GT‐pathway interaction (Figure 2E to G and Supporting information Table S6) and GT‐protein interaction networks (Supporting information Figures S8 to S10 and Table S7) were constructed, respectively. Similar functions were enriched, such as immune response and signal transduction, suggesting the cross‐talk between GTs and interacting proteins synergistically contributed to the biological alterations in cancer. In the interaction network, FBXO6 was the most common interacted protein, especially associated with KDELC2 and higher expression of KDELC2 and FBXO6 collectively contributed to the poor prognosis in LGG (Supporting information Figure S9). Further correlation analysis between GTs and tumor microenvironment (TME) was performed (Figure 3A). MFNG was significantly positively related to activated CD8^+^ T cells compared with other GTs, especially in LUAD and SKCM (Figure 3B and C), and higher expression of MFNG indeed conveyed better prognosis in LUAD and SKCM (Figure 3D). For melanoma patients treated with PD‐1 blockade,^6^ elevated MFNG also indicated a satisfactory prognosis (Figure 3E). Furthermore, MFNG was found to be mainly expressed in CX3CR1^+^ cytotoxic T cells based on two single‐cell RNA sequencing datasets^7^, ^8^ (Figure 3F and G). Considering previous studies,^9^ our findings of the expression of MFNG in CX3CR1^+^ cytotoxic T cells suggested that MFNG may be critically important in maintaining the function of this subset of CD8^+^ T cells. In addition to LUAD and SKCM, GTs correlating with the prognosis of patients in CD8^+^ T cell‐enriched tumors were observed in other 26 types of cancers (Supporting information Table S8).

**FIGURE 3:**
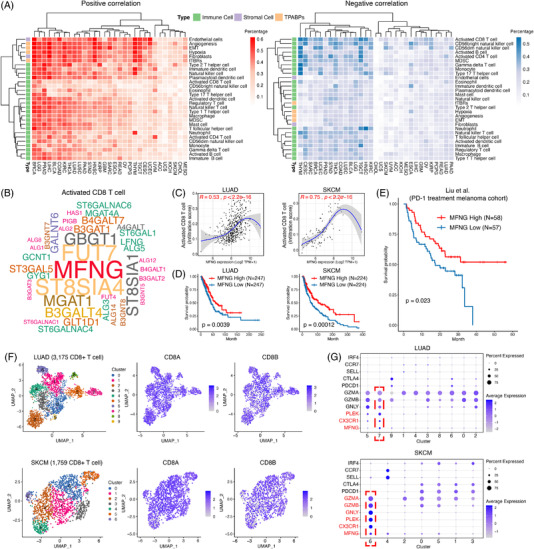
Identification of the association between MFNG and CD8^+^ T cell. (A) Proportions of GTs that were positively (left) or negatively (right) correlated with TME components across cancer types (*p* < 0.05). (B) Word cloud plot showing MFNG was significantly associated with activated CD8^+^ T cell. MFNG ranked the top in terms of the number of cancer types in which it was significantly positively related to activated CD8^+^ T cells. (C) Correlation analysis between MFNG and activated CD8^+^ T cell in LUAD (left) and SKCM (right). (D) Survival analysis of MFNG in LUAD (left) and SKCM (right). (E) Survival analysis of MFNG in melanoma patients with the intervention of PD‐1 blockade. (F) UMAP clustering of CD8^+^ T cells in LUAD (upper) and SKCM (lower), 3175 CD8^+^ T cells from LUAD and 1759 CD8^+^ T cells from SKCM. (G) Characteristic genes for each cluster (adjusted *p* < 0.05). In LUAD, MFNG is mainly expressed in cluster 7, while in SKCM, MFNG is mainly expressed in cluster 6. Both of the two clusters expressed CX3CR1 and the markers of cytotoxic T cells, including GZMA, GZMB, and GNLY, but they did not express the markers of exhausted T cells, such as PDCD1 and CTLA4, indicating the activation status of these T cells

A scoring tool (GTscore) was established using the expression of GTs and this score could reflect the tumor proliferation‐related activities, and predict the prognosis and treatment benefits of patients receiving immunotherapy (Supporting information Figure S11A and Table S9). In 16 cancer types, GTscore was associated with the prognosis of patients (Supporting information Figure S11B), and for immunotherapy cohort, patients with high GTscore displayed poorer prognosis and therapeutic disadvantages (Supporting information Figure S11C and D). High GTscore group showed higher levels of proliferation‐related activities, such as angiogenesis, EMT and hypoxia (Supporting information Figure S12).

The proliferation subgroup was an attractive clustering part of LIHC in our previous study.^10^ Here, GTs were found to be significantly correlated with proliferation‐related activities in LIHC (Figure 4A). Unsupervised consensus clustering based on the expression profiling of GTs could identify two clusters of LIHC patients, which showed different clinical outcomes and TME features (Figure 4B to D and Supporting information Figures S13 and S14). According to our proteogenomic resource of LIHC (CHCC‐HBV),^10^ GTs that contributed to different prognosis of patients were further analyzed. Among them, three GTs (GALNT4, MGAT5 and UGGT2) displayed prognosis relevance at protein level (Figure 4E and F). In addition, we found that the three GTs were highly expressed in the proliferation subtype (Figure 4G), therefore, according to this observation, these three GTs were chosen for further Tissue microarray (TMA) validation and cell‐based assays. The TMA comprising 154 cases showed patients with high MGAT5 or UGGT2 expression, indeed had shorter overall survival than patients with low expression (Figure 4H and I). Further analysis on the interacting proteins revealed that the expression of MGAT5 was correlated with ISLR, and the expression of UGGT2 was correlated with APP (Supporting information Figure S15). Transwell and CCK‐8 assays confirmed that downregulation of GALNT4, MGAT5 or UGGT2 could inhibit the migration and proliferation of LIHC cell lines (Figure 4J and K and Supporting information Figure S16).

**FIGURE 4:**
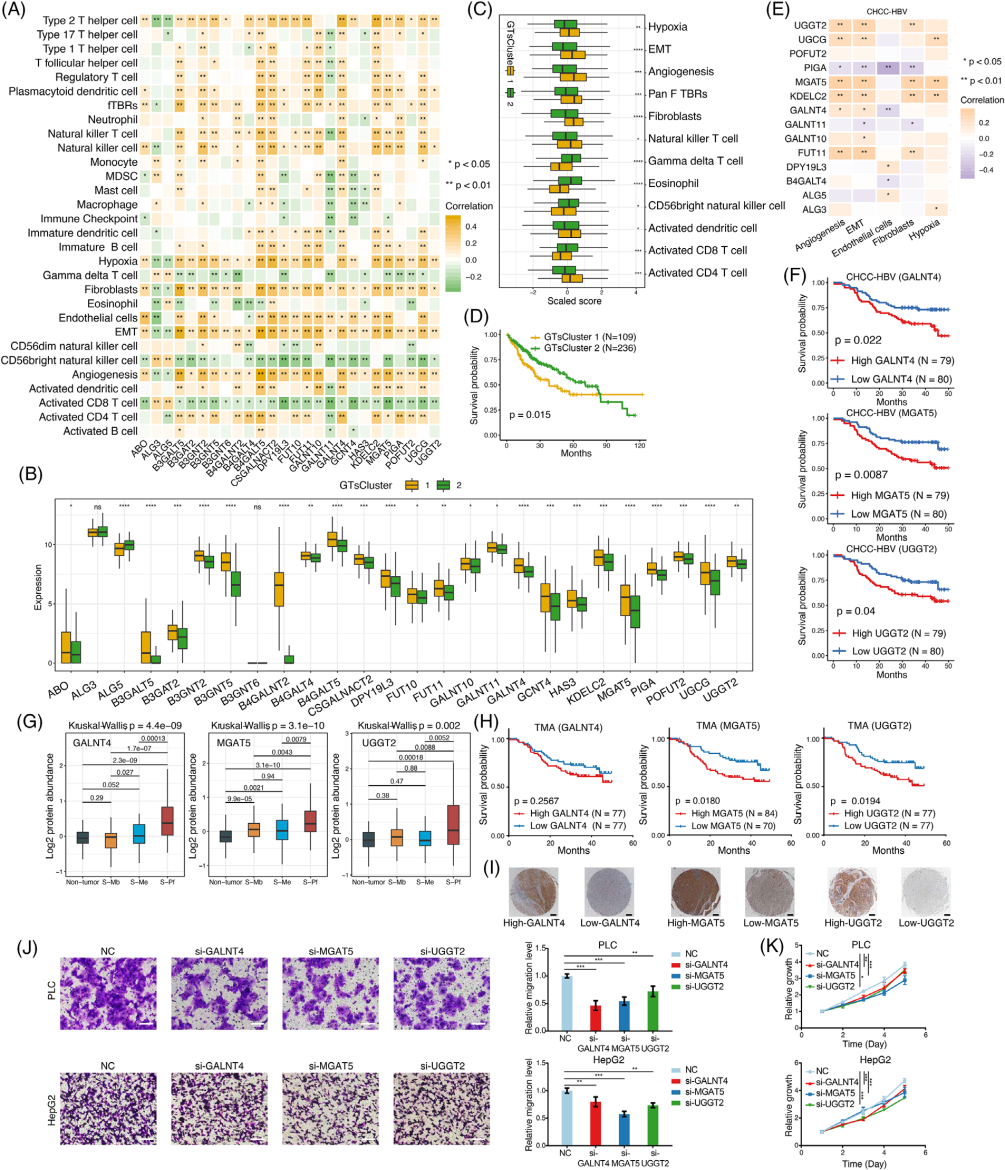
Identification and validation of GALNT4, MGAT5, and UGGT2 in LIHC. (A) Correlation between TME components and GTs that were significantly positively correlated with angiogenesis, EMT, hypoxia, and stromal cells in LIHC. (B) Two clusters were identified based on the expression of GTs (*p < 0.05; **p < 0.01; ***p < 0.001). (C) Comparison of tumor microenvironment features between GTsCluster 1 and GTsCluster 2 (*p < 0.05; **p < 0.01; ***p < 0.001). GTsCluster 1 was enriched in angiogenesis, EMT, hypoxia and fibroblasts, while GTsCluster 2 showed enrichment of CD8^+^ T cells, eosinophils and γδT cells. (D) Survival difference between GTsCluster 1 and GTsCluster 2. A prominent survival advantage was observed in GTsCluster 2. (E) Validation of GTs based on CHCC‐HBV cohort. GTs at protein level that were significantly associated with angiogenesis, EMT, hypoxia, and stromal cells in CHCC‐HBV cohort were shown. (F) Protein expressions of GALNT4, MAGT5, and UGGT2 were associated with patients’ survival in CHCC‐HBV cohort. (G) Protein abundance of GALNT4, MAGT5, and UGGT2 in different subtypes. These three GTs were highly expressed in the proliferation subtype. (H) Survival analysis of GALNT4, MGAT5, and UGGT2 and (I) typical IHC images based on TMAs. Scale bar: 200 μm. (J) Cell migration abilities in different GALNT4, MGAT5, and UGGT2 expression groups using transwell assay. Scale bar: 100 μm (**p <  0.01; ***p <  0.001). (K) Knockdown of GALNT4, MGAT5, and UGGT2 inhibited cell proliferation using CCK‐8 assay (statistical values obtained at the 5th day, *p < 0.05; **p < 0.01; ***p < 0.001)

Further validation of the biological implications of aberrantly expressed GTs, discovery of the common substrate of GTs and deciphering the site‐specific function of this substrate are necessary, and would provide vital clues for the diagnosis or treatment of cancers via targeting specific glycosylation.

## FUNDING INFORMATION

The work was supported by Shanghai Pujiang Program (2020PJD012), National Natural Science Foundation of China (82150111, 91859105 and 81961128025) and the Science and Technology Commission of Shanghai Municipality (20JC1418900).

## CONFLICT OF INTEREST

The authors declare that they have no competing interest.

## Supporting information

Supporting Information
