# Supplementary material for: An integrative pan‐cancer analysis of the molecular and biological features of glycosyltransferases
Source: Clin Transl Med. 2022 Jul 8;12(7):e872. doi: 10.1002/ctm2.872 (PMC9270580; doi:10.1002/ctm2.872)
Supplement: Supplementary file 1 — Supporting Information [file CTM2-12-e872-s001.zip › ctm2872-sup-0001-SuppMat/SupplementaryFiles20220516/SFigure/S8.pdf]

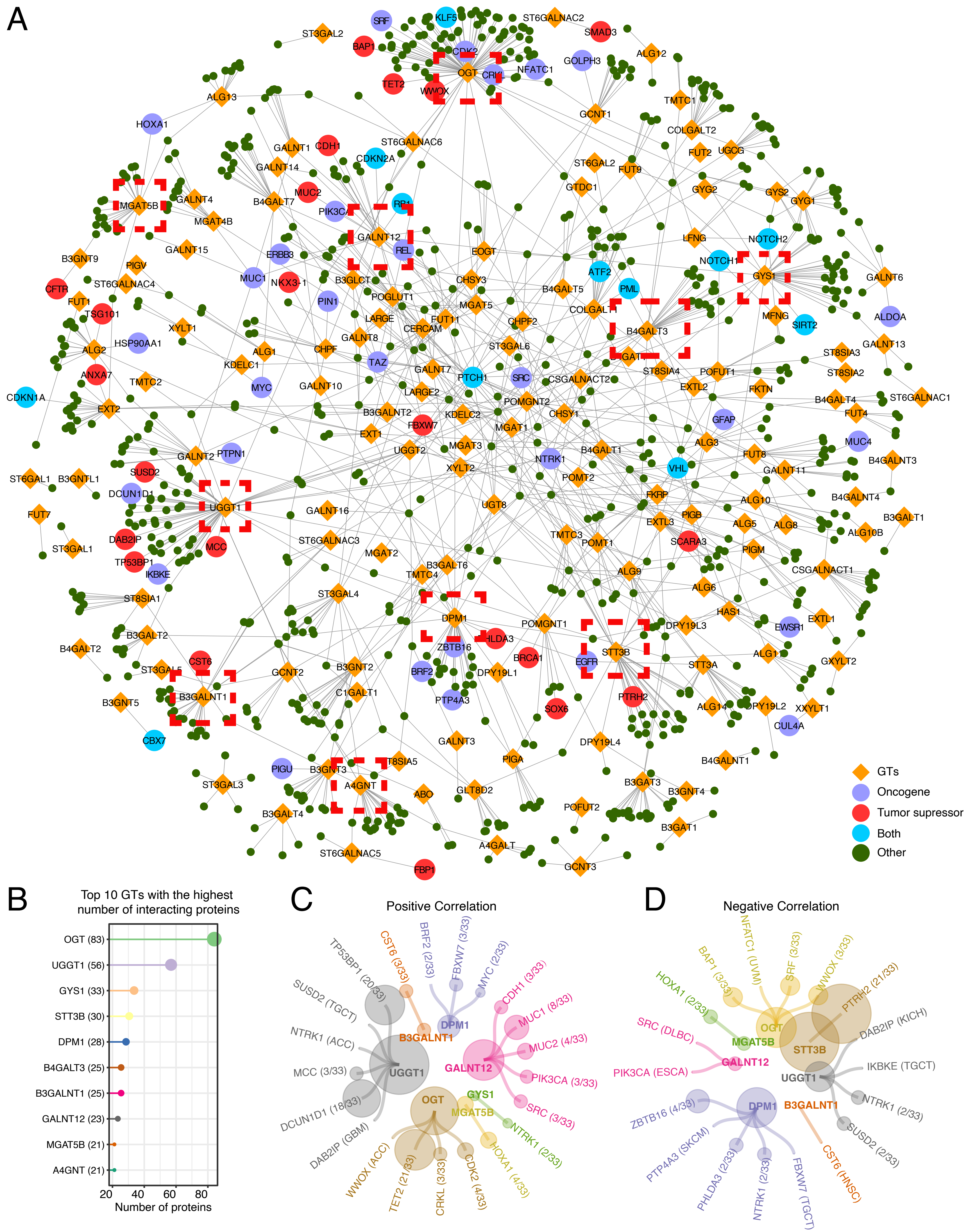

A
GTs
Oncogene
Tumor supressor
Both
Other
ST6GALNAC2
SRF
KLF5
SMAD3
ST3GAL2
BAP1
CDK2
ALG12
GOLPH3
OGT
CRKL
NFATC1
TET2
WWOX
TMTC1
ALG13
GCNT1
HOXA1
COLGALT2
GALNT1
CDH1
ST6GALNAC6
FUT2
UGCG
CDKN2A
ST6GAL2
FUT9
GALNT14
GTDC1
MUC2
GYG2
GYS2
GYG1
MGAT5B
GALNT4
B4GALT7
RB1
PIK3CA
MGAT4B
GALNT12
LFNG
NOTCH2
B3GNT9
GALNT15
ERBB3
REL
EOGT
NOTCH1
PIGV
GYS1
CFTR
MUC1
NKX3-1
B3GLCT
CHSY3
ATF2
GALNT6
ST6GALNAC4
POGLUT1
B4GALT5
PML
FUT1
PIN1
MGAT5
TSG101
LARGE
FUT11
CHPF2
COLGALT1
MFNG
ALDOA
CERCAM
SIRT2
XYLT1
B4GALT3
ALG2
HSP90AA1
GALNT8
ALG1
CHPF
ST3GAL6
GALNT13
ST8SIA3
KDELC1
TAZ
ANXA7
GALNT7
PTCH1
SRC
ST8SIA4
POFUT1
ST8SIA2
TMTC2
CSGALNACT2
MYC
GALNT10
EXTL2
FKTN
CDKN1A
LARGE2
EXT2
POMGNT2
B4GALT4
ST6GALNAC1
B3GALNT2
KDELC2
CHSY1
FUT4
GFAP
FBXW7
EXT1
MGAT1
ALG3
MUC4
UGGT2
MGAT3
B4GALT1
GALNT2
PTPN1
FUT8
NTRK1
B4GALNT3
XYLT2
VHL
GALNT11
ST6GAL1
B3GNTL1
SUSD2
POMT2
FKRP
DCUN1D1
ALG10
UGT8
B4GALNT4
FUT7
UGGT1
GALNT16
PIGB
B3GALT1
EXTL3
ALG5
ALG8
TMTC3
ALG10B
DAB2IP
ST6GALNAC3
POMT1
SCARA3
ST3GAL1
MCC
MGAT2
PIGM
TP53BP1
B3GALT6
CSGALNACT1
IKBKE
TMTC4
ALG9
ST8SIA1
ST3GAL4
ALG6
HAS1
DPM1
EXTL1
POMGNT1
DPY19L3
EWSR1
B3GALT2
PHLDA3
B4GALT2
ZBTB16
STT3B
ALG11
CST6
BRCA1
EGFR
STT3A
GXYLT2
ST3GAL5
GCNT2
B3GNT2
BRF2
DPY19L1
B3GALNT1
C1GALT1
PTRH2
ALG14
DPY19L2
XXYLT1
B3GNT5
PTP4A3
SOX6
CUL4A
CBX7
GALNT3
DPY19L4
PIGA
ST8SIA5
B4GALNT1
PIGU
B3GNT3
A4GNT
ABO
ST3GAL3
GLT8D2
B3GAT3
B3GNT4
POFUT2
B3GALT4
B3GAT1
A4GALT
ST6GALNAC5
FBP1
GCNT3
B
Top 10 GTs with the highest number of interacting proteins
OGT (83)
UGGT1 (56)
GYS1 (33)
STT3B (30)
DPM1 (28)
B4GALT3 (25)
B3GALNT1 (25)
GALNT12 (23)
MGAT5B (21)
A4GNT (21)
20
40
60
80
Number of proteins
C
Positive Correlation
TP53BP1 (20/33)
CST6 (3/33)
BRF2 (2/33)
FBXW7 (3/33)
MYC (2/33)
CDH1 (3/33)
SUSD2 (TGCT)
DPM1
MUC1 (8/33)
B3GALNT1
NTRK1 (ACC)
MUC2 (4/33)
GALNT12
UGGT1
MCC (3/33)
PIK3CA (3/33)
GYS1
DCUN1D1 (18/33)
OGT
MGAT5B
SRC (3/33)
NTRK1 (2/33)
DAB2IP (GBM)
HOXA1 (4/33)
WWOX (ACC)
TET2 (21/33)
CRKL (3/33)
CDK2 (4/33)
D
Negative Correlation
NFATC1 (UVM)
BAP1 (3/33)
SRF (3/33)
WWOX (3/33)
HOXA1 (2/33)
PTRH2 (21/33)
OGT
SRC (DLBC)
MGAT5B
STT3B
DAB2IP (KICH)
GALNT12
PIK3CA (ESCA)
UGGT1
IKBKE (TGCT)
DPM1
B3GALNT1
ZBTB16 (4/33)
NTRK1 (2/33)
SUSD2 (2/33)
PTP4A3 (SKCM)
PHLDA3 (2/33)
NTRK1 (2/33)
FBXW7 (TGCT)
CST6 (HNSC)
