# Supplementary material for: An integrative pan‐cancer analysis of the molecular and biological features of glycosyltransferases
Source: Clin Transl Med. 2022 Jul 8;12(7):e872. doi: 10.1002/ctm2.872 (PMC9270580; doi:10.1002/ctm2.872)
Supplement: Supplementary file 1 — Supporting Information [file CTM2-12-e872-s001.zip › ctm2872-sup-0001-SuppMat/SupplementaryFiles20220516/SupplementaryFiles.docx]

**Supplementary Information**

**An integrative pan-cancer analysis of the molecular and biological features of glycosyltransferases**

Yin Li^1,5^, Youpei Lin^2,3,5^, Ling Aye^2,5^, Liangqing Dong^2^, Chenhao Zhang^2^, Fanghua Chen^2^, Yinkun Liu^2,3^, Jia Fan^2,3^, Qiang Gao^2,3^, Haojie Lu^3,4,*^, Chunlai Lu^1,*^, Shu Zhang^2,3,*^

^1^Department of Thoracic Surgery, Zhongshan Hospital, Fudan University, Shanghai, P.R. China

^2^Liver Cancer Institute, Zhongshan Hospital, and Key Laboratory of Carcinogenesis and Cancer Invasion (Ministry of Education), Fudan University, Shanghai, P.R. China

^3^Institutes of Biomedical Sciences, Fudan University, Shanghai, P.R. China

^4^NHC Key Laboratory of Glycoconjugates Research and Department of Chemistry, Fudan University, Shanghai, P.R. China

^5^These authors contributed equally

*Corresponding author

**Correspondence**

Shu Zhang, E-mail: zhang.shu@zs-hospital.sh.cn

Chunlai Lu, E-mail: lu.chunlai@zs-hospital.sh.cn

Haojie Lu, E-mail: luhaojie@fudan.edu.cn

Abbreviations
ACC, Adrenocortical Carcinoma; BLCA, Bladder Urothelial Carcinoma; BRCA, Breast Invasive Carcinoma; CESC, Cervical Squamous Cell Carcinoma and Endocervical Adenocarcinoma; CHOL, Cholangio Carcinoma; COAD, Colon Adenocarcinoma; DLBC, Lymphoid Neoplasm Diffuse Large B-cell Lymphoma; ESCA, Esophageal Carcinoma; GBM, Glioblastoma Multiforme; HNSC, Head and Neck Squamous Cell Carcinoma; KICH, Kidney Chromophobe; KIRC, Kidney Renal Clear Cell Carcinoma; KIRP, Kidney Renal Papillary Cell Carcinoma; LAML, Acute Myeloid Leukemia; LGG, Brain Lower Grade Glioma; LIHC, Liver Hepatocellular Carcinoma; LUAD, Lung Adenocarcinoma; LUSC, Lung Squamous Cell Carcinoma; MESO, Mesothelioma; OV, Ovarian Serous Cystadenocarcinoma; PAAD, Pancreatic Adenocarcinoma; PCPG, Pheochromocytoma and Paraganglioma; PRAD, Prostate Adenocarcinoma; READ, Rectum Adenocarcinoma; SARC, Sarcoma; SKCM, Skin Cutaneous Melanoma; STAD, Stomach Adenocarcinoma; TGCT, Testicular Germ Cell Tumors; THCA, Thyroid Carcinoma; THYM, Thymoma; UCEC, Uterine Corpus Endometrial Carcinoma; UCS, Uterine Carcinosarcoma; UVM, Uveal Melanoma. TCGA, The Cancer Genome Atlas; CCLE, Cancer Cell Line Encyclopedia.

**Supplementary Materials and Methods**

**Data acquisition and processing**

A total of 185 GTs were collected from the previously published study **(Table S1)**(1). Based on the carbohydrate-active enzymes (CAZy) database ([www.cazy.org](http://www.cazy.org)), GTs were classified into different categories. Specifically, 27 GTs are involved in N-glycosylation, 26 in O-GalNAc, 9 in Glycosphingolipid, 3 in O-Glucose, 16 in Glycosaminoglycan, 2 in O-GlcNAc, 6 in O-Fucose, 15 in O-Mannose, 4 in C-Mannose, 2 in O-Galactose, 3 in Hyaluronan, 4 in Glycogen, 5 in GPI anchor, 19 in Elongation, 35 in Capping, and 9 in unspecified (Other) ones.

The Cancer Genome Atlas (TCGA) multi-omics data were downloaded from Xena public data hubs (<https://xenabrowser.net>), including somatic mutation (Gene level non-silent mutation, Pan-Cancer Atlas Hub), copy number variation (gene-level copy number, GISTIC2_thresholded, TCGA Hub), gene expression (Batch effects normalized mRNA data, Pan-Cancer Atlas Hub), phenotype and clinical information (Curated clinical data, Pan-Cancer Atlas Hub). Cancer cell line mutation and drug resistance data were collected from the Broad Institute Cancer Cell Line Encyclopedia (CCLE)(2).

Single-cell RNA sequencing data of lung adenocarcinoma (GSE131907) and melanoma (GSE115978) samples from the initial publications were downloaded and reanalyzed(3, 4). CD8^+^ T cells were extracted based on the annotations of initial publications. Seurat (v4.0.0) workflow was then performed(5). The FindMarkers function in Seurat was used to identify differentially expressed genes for each cluster (adjusted p-value < 0.05).

Two expression profiles of patients receiving immune checkpoint blockade (ICB) were obtained, including advanced urothelial cancer patients with the intervention of anti-PD-L1 antibody (IMvigor210 cohort), and melanoma patients treated with anti-PD-1 therapy(6, 7). The expression values were uniformly processed and converted to TPM for further analysis.

**Somatic mutation and copy number variation analysis**

The mutation frequency of each GT was defined as the proportion of tumor samples burdened with the mutation of the given GT. The mutation frequency of each GT in each cancer cell line was defined as the proportion of cell lines with the mutations of the given GT. Only the non-silent mutations (Missense_Mutation, In_Frame_Del, Nonsense_Mutation, Splice_Site, In_Frame_Ins, Frame_Shift_Del, Nonstop_Mutation, Frame_Shift_Ins, Translation_Start_Site, and Large deletion) were included for mutation frequency calculation. For copy number variations (CNV) analyses, GISTIC2 thresholded gene-level data were used(8). The GISTIC2 thresholded data provided five levels of CNV, that is, homozygous deletion (-2), single copy deletion (-1), diploid normal copy (0), low-level copy number amplification (+1) and high-level copy number amplification (+2). In this analysis, genes with positive values of +1 and +2 were considered CNV gain, whereas genes assigned negative values of -1 and -2 were considered CNV loss. We defined the CNV frequency as the proportion of samples with CNV gain or loss in a given cancer type. For CCLE CNV data, we calculated the CNV frequency as the proportion of cell lines with CNV gain and loss. The difference between the frequency of CNV gain and loss was calculated, and an absolute value of 0.3 was used as the cut-off value.

**Gene expression, GTs-pathway interaction network, and protein interaction analyses**

Unsupervised hierarchical clustering of the expression profiling of GTs was conducted using *hclust* package to explore whether different types of cancer would show similar expression features of particular GTs. Differential expression analysis was performed using wilcoxon rank sum test, p-values were adjusted using BH method. Genes with adjusted p-values < 0.05 were identified as differentially expressed genes. To infer the potential biological functions of GTs, a pan-cancer GTs-pathway interaction landscape was constructed. Within each cancer type, we utilized the bicor function from WGCNA R package to calculate the transcriptomics-based biweight midcorrelations between each GT and all the other genes(9). We then ranked all genes based on the absolute values. Top 500 genes were used to identify the pathways significantly impacted by GTs using signaling pathway impact analysis (SPIA) algorithm(10). The pathway information was obtained from the KGML files from KEGG database (https://www.kegg.jp/kegg/pathway.html). To systematically evaluate the GTs associated protein interaction network, we used BisoGenet application of Cytoscape v3.7.2 to construct the protein-protein interaction network. DIP, BIOGRID, HPRD, INTACT, MINT and BIND databases were retrieved for the PubMed supported GTs-protein interaction network. The proteins used for the network construction was previously reported with at least one glycosite based on UniPort database (<https://www.uniprot.org/>).

**TME components and related biological processes estimation**

ssGSEA (single sample gene set enrichment analysis) method was used to quantify the relative abundance of each cell infiltration in the TME. The gene set for marking each TME infiltration immune cell type was obtained from the previously published study, which enabled us to quantify various immune cell subtypes including activated CD8^+^ T cell, macrophage, natural killer T cell and regulatory T cell(11). The enrichment scores calculated through ssGSEA analysis were used to represent the abundance of each infiltrating cell in each sample. The stroma cell populations were calculated based on MCPcounter method(12). The tumor proliferation-associated biological processes were quantified using GSVA method, and the gene sets were collected from previously published study.

**Prognosis analysis of GTs in CD8^+^ T cell-enriched samples**

CD8^+^ T cells abundance was inferred using MCPcounter(12). The median value was used as the cut-off value. Survival analysis of GTs in tumor samples with a higher abundance of CD8^+^ T cells was conducted.

**Construction of GTscore**

The GTs-based gene signature was constructed as follows. First, we kept the GTs that were found related to patients’ survival in at least one cancer type based on the univariate survival analysis. Then, the kept GTs were fitted into LASSO Cox regression analysis in the pan-cancer cohort to reduce dimensionality and identify representative makers. Ten-fold cross-validation was used to determine the values of λ and we chose the λ where the partial likelihood deviance is the smallest. According to the λ value, each remaining GT was assigned with a LASSO coefficient, and GTscore was generated using the following formula mentioned previously(13). Survminer R package was used to determine the optimal cut-off point for classifying high and low score groups based on the maximally selected log-rank statistics(14).

**GTs related subtypes identification in LlHC**

Unsupervised consensus clustering was conducted to cluster tumor samples into subgroups based on the expression matrix of GTs using ConsensusClusterPlus R package(15). The parameters were used: number of repetitions = 1,000 bootstraps; pItem = 0.8 (resampling 80% of any sample); pFeature = 0.8 (resampling 80% of any protein); and clustering algorithm = k-means method. The clustering that exhibited the most significant survival difference was considered.

**Clinical samples and tissue microarrays**

Tissue microarrays (TMAs) of 154 tumor tissues from the CHCC-HBV cohort were collected as previously described to examine the expression and clinical values of GALNT4, MGAT5, and UGGT2(16). Sides were rehydrated, antigen retrieved, blocked and incubated with primary antibodies (GALNT4: 12897-1-AP, Proteintech; MGAT5: bs-5841R, Bioss; UGGT2: 13420-1-AP, Proteintech). After 12 hours, slides were treated with secondary antibodies (Genetech, Shanghai) and colored by DAB peroxidase substrate kit (Genetech, Shanghai) for immunohistochemistry (IHC). The staining levels were quantitated independently by two observers. Based on the staining intensities of target proteins in each case, a score of 0-2 was classified as low expression, a score of 3-4 was classified as medium expression, and a score of 5-6 was classified as high expression. Finally, patients were classified into high and low expression groups by comparing its score with the median value.

**Cell culture and transfection**

HepG2 and PLC cell lines were purchased from the Chinese Academy of Science Cell Bank (Shanghai, China). All cell lines were maintained in DMEM (Hyclone, USA) with 10% FBS (Gibco, USA), 100 units of penicillin and 100 μg/mL streptomycin (Hyclone, USA) in a humidified incubator with 5% CO_2_.

**RNA Interference**

The small interfering RNAs (siRNAs) were synthesized by Shanghai GenePharma Co., Ltd., (Shanghai, China) and transfected with Hieff Trans™ Liposomal Transfection Reagent (Yeasen, China) according to the manufacture’s instruction. The siRNA transfected cells were harvested for further assays 48 hours after transfection. Oligonucleotide sequences are as following:

siUGGT2 Sense: 5′- GCCUCAAGCUCUUUAUAAUTT-3′, Antisense:5′-AUUAUAAAGAGCUUGAGGCTT-3′;

siMGAT5 Sense: 5′- CCUGGAAGCUAUCGCAAAUTT-3′, Antisense:5′-AUUUGCGAUAGCUUCCAGGTT-3′;

siGALNT4: Sense: 5′- GCCGACCUGAUGUACAAAUTT-3′, Antisense:5′-AUUUGUACAUCAGGUCGGCTT-3′.

**CCK-8 (cell counting kit-8) assay**

Cells were plated in 96-well plates at a density of 2 × 10^3^ cells/well and cell viability was investigated after 24, 48, 72, 96, and 120 hours. CCK-8 solution (Dojindo, Japan) was added to each well. After 120 min, the absorbance at 450 nm was recorded.

**Cell migration assay**

Migration assay was performed in transwell chambers (Corning, USA). Cells were seeded onto the upper chambers at a density of 6-10 × 10^4^/well; the lower chamber was filled with 800 μl DMEM medium containing 10% FBS, and 100 units of penicillin and 100 μg/mL streptomycin. After incubation, the cells on the bottom side of the filter were fixed in 4% paraformaldehyde, stained with 0.1% crystal violet, and were quantified under a light microscope. Results were obtained from three independent experiments.

**RT-PCR (Real-time PCR)**

Total RNA was purified using RNA Extraction Reagent Kit (Yeasen, China) and cDNA was synthesized using the cDNA Synthesis Kit (Yeasen, China) according to the manufacturer’s instructions. RT-PCR was performed using SYBR Green Realtime PCR Master Mix (Yeasen, China). The primer sequences used in this study were as follows:

GAPDH: GGACTCATGACCACAGTCCA (F) & CCAGTAGAGGCAGGGATGAT (R);

MGAT5: CTTCACTCCGTGGAAGTTGTC (F) & TGGATGGTAAAGTGCAGAAGC (R);

GALNT4: ACAGTGGCCTATATCTTCGTGG (F) & CTCCTGCGGAGGCATGAAAA (R);

UGGT2: CCTTCGCAATCTTGGGATCAA (F) & GCCGGATCAATAAACAGAACCA (R).

**Statistical analysis**

Correlation analysis was conducted using the Spearman method. Pearson’s chi-square analysis was used to calculate composition differences. The statistical difference of the two groups was calculated using the Wilcoxon rank sum test. For comparisons of more than two groups, the Kruskal-Wallis test was used. Experimental results were expressed as mean ± standard error of the mean (SEM). For trans-well assay, Kruskal-Wallis test was used to test if any of the differences between the subgroups were statistically significant. For CCK-8 assay, the statistical significance of differences was determined by two-way ANOVA. For RT-PCR, the statistical significance of differences was determined by t-test. Each experiment was conducted in triplicate. Log-rank test was used to describe overall survival. All statistical tests were two-sided, and statistical significance was considered when p-value < 0.05. Statistical analysis was conducted using R and GraphPad Prism.

**Data availability**

The Cancer Genome Atlas (TCGA) multi-omics data were downloaded from Xena public data hubs (<https://xenabrowser.net>). Cancer cell line mutation and drug resistance data were collected from the Broad Institute Cancer Cell Line Encyclopedia (CCLE). Other publicly available data were described in the Method section.

**Supplementary References**

1. Narimatsu Y, Joshi HJ, Yang Z, Gomes C, Chen YH, Lorenzetti FC, et al. A validated gRNA library for CRISPR/Cas9 targeting of the human glycosyltransferase genome. Glycobiology. 2018;28(5):295-305.

2. Ghandi M, Huang FW, Jane-Valbuena J, Kryukov GV, Lo CC, McDonald ER, 3rd, et al. Next-generation characterization of the Cancer Cell Line Encyclopedia. Nature. 2019;569(7757):503-8.

3. Kim N, Kim HK, Lee K, Hong Y, Cho JH, Choi JW, et al. Single-cell RNA sequencing demonstrates the molecular and cellular reprogramming of metastatic lung adenocarcinoma. Nat Commun. 2020;11(1):2285.

4. Jerby-Arnon L, Shah P, Cuoco MS, Rodman C, Su MJ, Melms JC, et al. A Cancer Cell Program Promotes T Cell Exclusion and Resistance to Checkpoint Blockade. Cell. 2018;175(4):984-97 e24.

5. Hao Y, Hao S, Andersen-Nissen E, Mauck WM, Zheng S, Butler A, et al. Integrated analysis of multimodal single-cell data. bioRxiv. 2020:2020.10.12.335331.

6. Mariathasan S, Turley SJ, Nickles D, Castiglioni A, Yuen K, Wang Y, et al. TGFbeta attenuates tumour response to PD-L1 blockade by contributing to exclusion of T cells. Nature. 2018;554(7693):544-8.

7. Liu D, Schilling B, Liu D, Sucker A, Livingstone E, Jerby-Arnon L, et al. Integrative molecular and clinical modeling of clinical outcomes to PD1 blockade in patients with metastatic melanoma. Nat Med. 2019;25(12):1916-27.

8. Mermel CH, Schumacher SE, Hill B, Meyerson ML, Beroukhim R, Getz G. GISTIC2.0 facilitates sensitive and confident localization of the targets of focal somatic copy-number alteration in human cancers. Genome Biol. 2011;12(4):R41.

9. Langfelder P, Horvath S. WGCNA: an R package for weighted correlation network analysis. BMC Bioinformatics. 2008;9:559.

10. Tarca AL, Draghici S, Khatri P, Hassan SS, Mittal P, Kim JS, et al. A novel signaling pathway impact analysis. Bioinformatics. 2009;25(1):75-82.

11. Bindea G, Mlecnik B, Tosolini M, Kirilovsky A, Waldner M, Obenauf AC, et al. Spatiotemporal dynamics of intratumoral immune cells reveal the immune landscape in human cancer. Immunity. 2013;39(4):782-95.

12. Becht E, Giraldo NA, Lacroix L, Buttard B, Elarouci N, Petitprez F, et al. Estimating the population abundance of tissue-infiltrating immune and stromal cell populations using gene expression. Genome Biol. 2016;17(1):218.

13. Li Y, Gu J, Xu F, Zhu Q, Chen Y, Ge D, et al. Molecular characterization, biological function, tumor microenvironment association and clinical significance of m6A regulators in lung adenocarcinoma. Brief Bioinform. 2020.

14. Zhou R, Zeng D, Zhang J, Sun H, Wu J, Li N, et al. A robust panel based on tumour microenvironment genes for prognostic prediction and tailoring therapies in stage I-III colon cancer. EBioMedicine. 2019;42:420-30.

15. Wilkerson MD, Hayes DN. ConsensusClusterPlus: a class discovery tool with confidence assessments and item tracking. Bioinformatics. 2010;26(12):1572-3.

16. Gao Q, Zhu H, Dong L, Shi W, Chen R, Song Z, et al. Integrated Proteogenomic Characterization of HBV-Related Hepatocellular Carcinoma. Cell. 2019;179(2):561-77 e22.

**Supplementary Figures**

**Supplementary Figure S1.** The impacts of mutations of GTs on gene expression and drug sensitivity. (A) Comparison of the expression level of GTs (mutation frequencies > 5%) in the mutation group vs. wild type group. (B) Mutations of FUT9 and GALNT13 in SKCM were linked to different drug sensitivities to certain drugs based on CCLE data.

**Supplementary Figure S2.** (A) DPY19L4, ST3GAL1, EXT1, HAS2, and POFUT1 showed widespread CNVs gains (Left). ST8SIA3, STT3B, EOGT, GXYLT2, and GLT8D1 displayed prevalent CNVs losses (right). (B) Spearman correlation between CNVs and gene expression of GTs. The expression of EXT1, DPY19L4, POFUT1, STT3B, and GLT8D1 significantly positively correlated with CNVs.


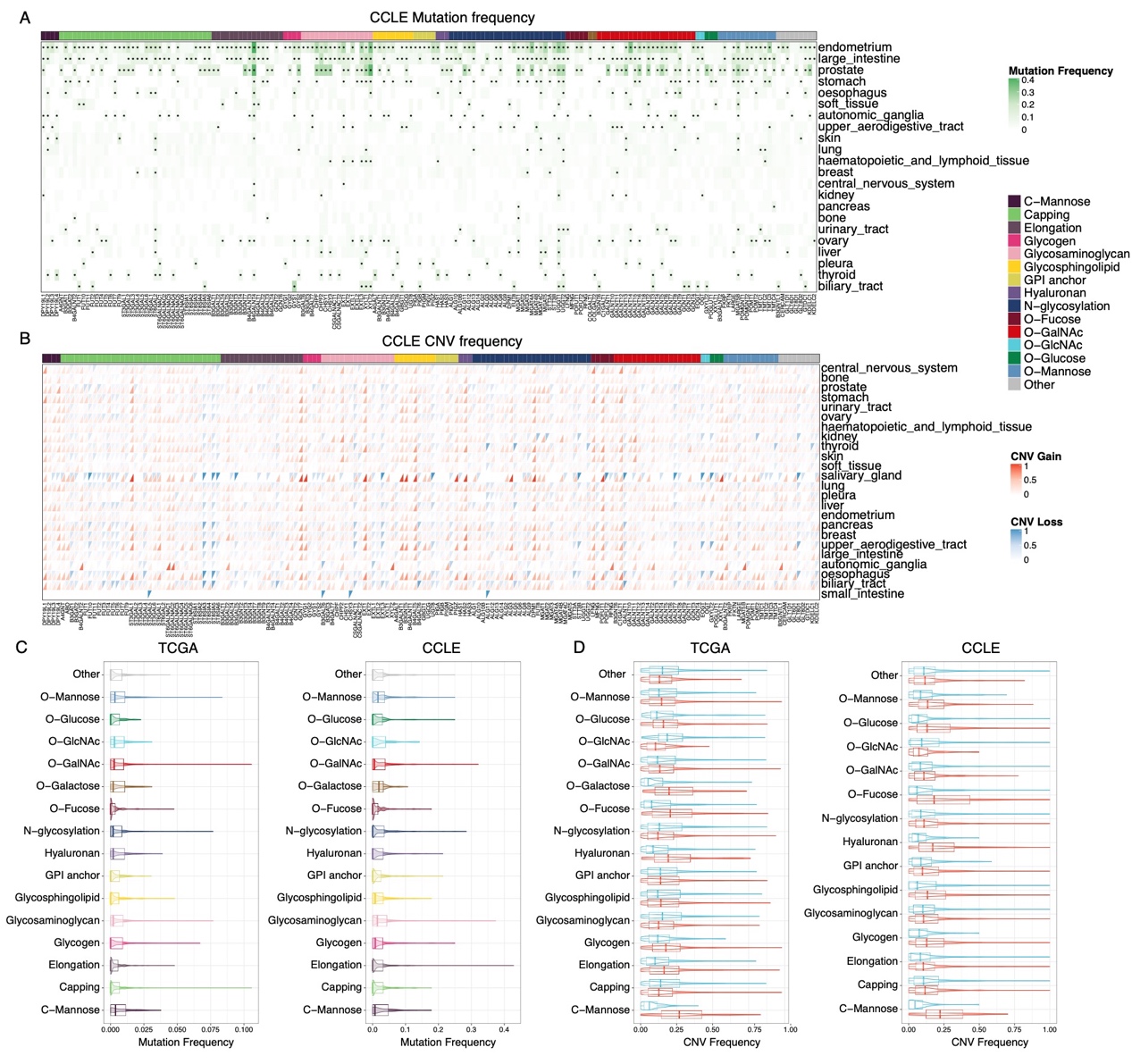


**Supplementary Figure S3.** Mutation frequency distribution and CNVs of GTs across cancer cell lines. (A) Mutation frequency of GTs across cancer cell lines. GTs with mutation frequency > 5% in any cancer cell line were highlighted as asterisks. (B) CNVs frequency of GTs across cancer cell lines. (C) Comparison of the distribution of mutation frequency of GTs between TCGA and CCLE. (D) Comparison of the distribution of CNVs frequency of GTs between TCGA and CCLE.


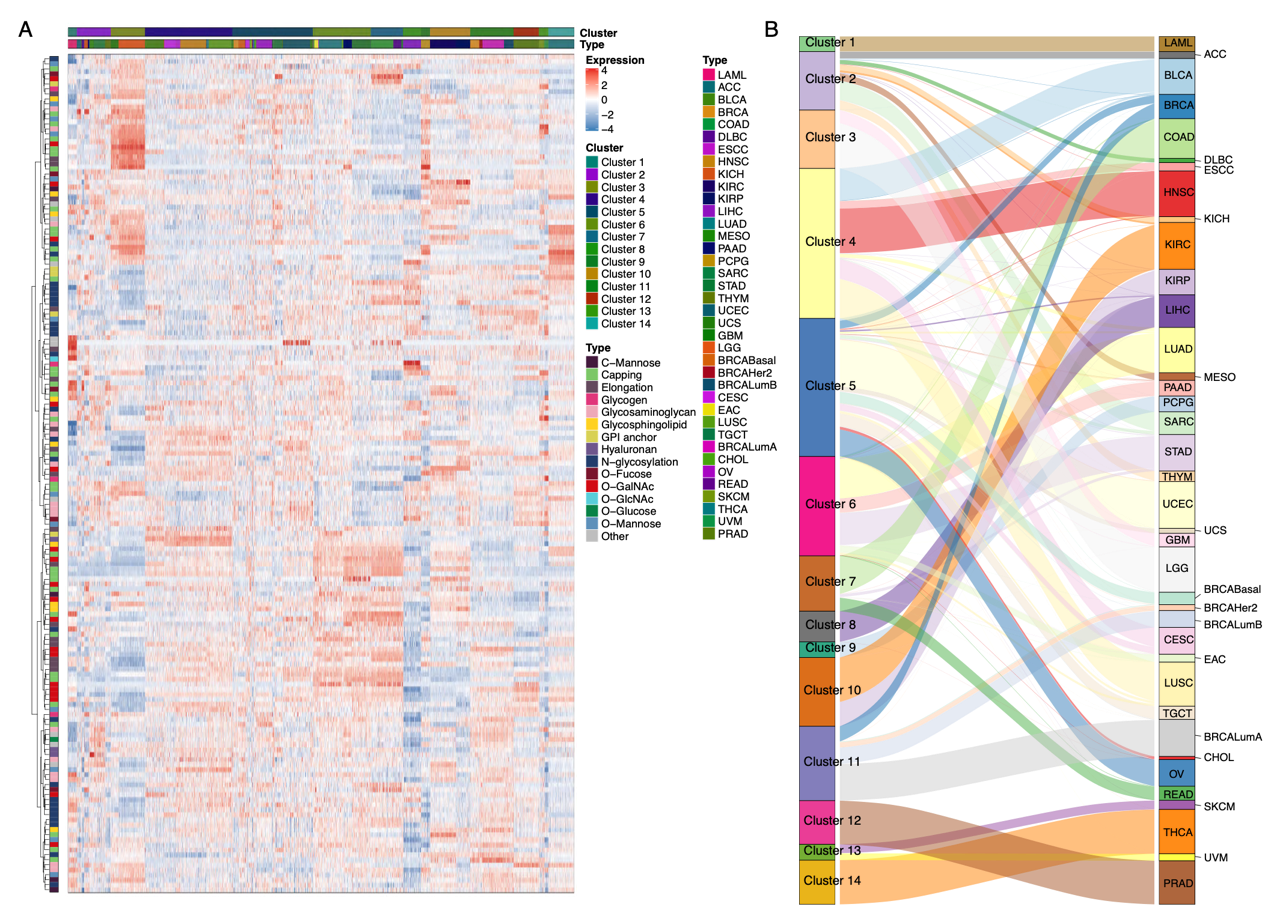


**Supplementary Figure S4.** Unsupervised hierarchical clustering of cancer samples based on the expression of GTs. (A) 14 major clusters were identified. (B) Composition of each cluster. Cancer types with squamous morphology (BLCA, CESC, ESCC, HNSC, and LUSC) grouped together in cluster 4. Cancer types that have glandular characteristics (adenocarcinomas) displayed similarities, including LUAD, PAAD, STAD, and EAC (cluster 6). On the other hand, some cancer types with tissue or organ similarities were grouped together. These included the lower digestive system (COAD and READ, cluster 7), clear cell and papillary renal carcinomas (KIRC and KIRP, cluster 10), and melanomas of the skin and eye (SKCM and UVM, cluster 13). This indicated that the expression patterns of GTs were associated with cancer morphology.

**Supplementary Figure S5.** Transcriptional features of GTs across cancer types. (A) Differential expression of GTs in different cancers. The color represents the fold changes. Asterisk indicated significantly differentially expressed GTs (adjusted p < 0.05). The red bar on the top represented the number of cancers in which the GT was found to be up-regulated. The blue bar on the top represented the number of cancers in which the GT found to be down-regulated. The red bar on the right represented the number of over-expressed GTs in each cancer. The red bar on the right represented the number of down-regulated GTs in each cancer. (B) Survival relevance of GTs. The color represented the hazard ratio. Asterisk indicated significantly prognosis related GTs (p < 0.05). The red bar on the top represented the number of cancers in which the GT found to be risky (hazard ratio > 1). The blue bar on the top represented the number of cancers in which the GT was found to be protective (hazard ratio < 1). The red bar on the right represented the number of risky GTs in each cancer. The red bar on the right represented the number of protective GTs in each cancer.


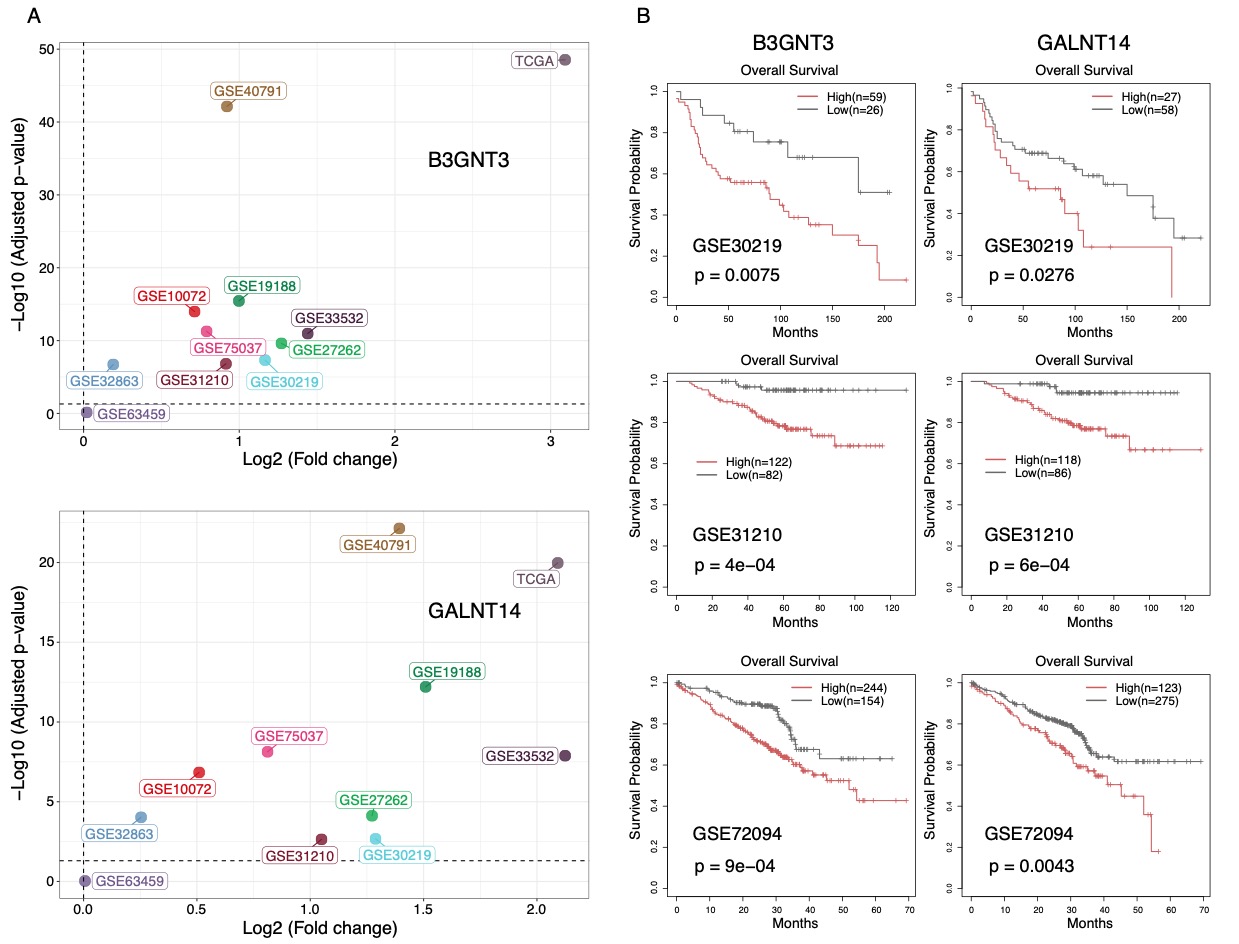


**Supplementary Figure S6.** Validation of B3GNT3 and GALNT14 in external LUAD cohorts based on LUADEXPRESS (http://www.bioinfo-zs.com/luadexpress/). (A) Expression alterations of B3GNT3 and GALNT14 in external LUAD datasets. (B) Survival analysis of B3GNT3 and GALNT14. The cut-off value was determined based on the maximally separated log-rank test.


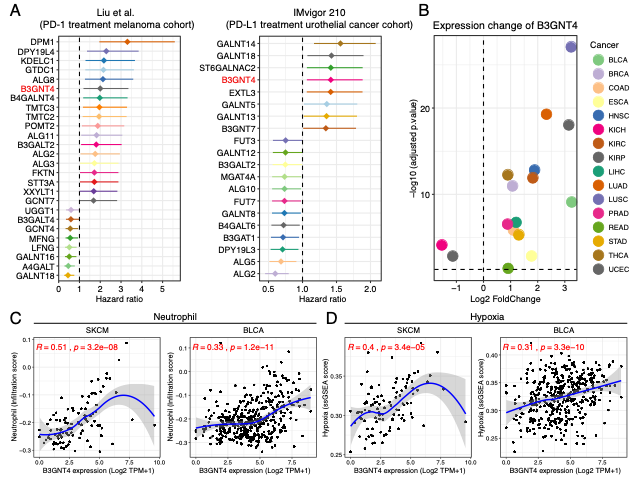


**Supplementary Figure S7.** Identification of B3GNT4 as an unfavorable factor for immunotherapy. (A) Survival analysis of GTs in Liu et al. and IMvigor210 cohorts. A GT with hazard ratio (HR) > 1 was defined as the risky GT, whereas HR < 1 as the protective GT (p < 0.05). (B) Expression alterations of B3GNT4 across cancer types. (C) Correlation of B3GNT4 with neutrophils. (D) Correlation of B3GNT4 with hypoxia.


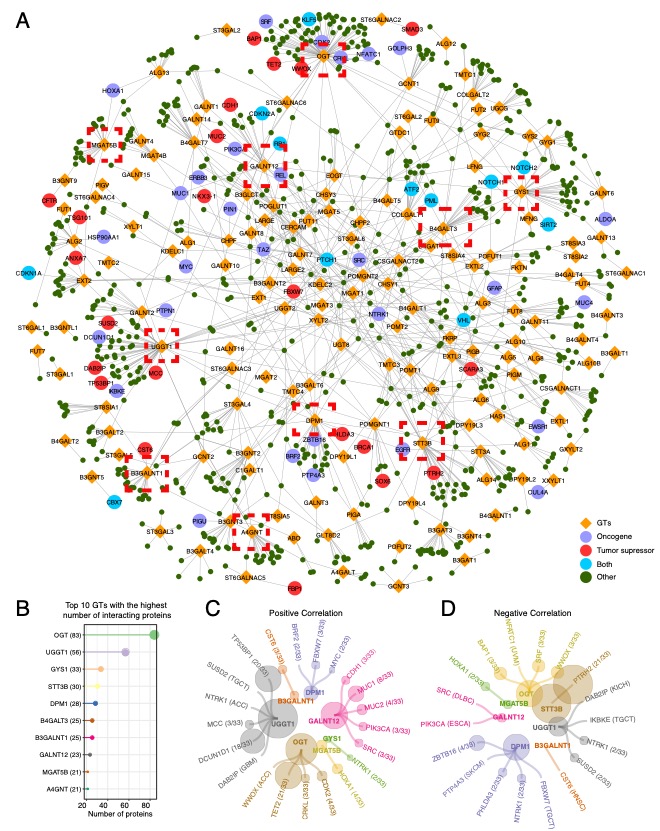


**Supplementary Figure S8.** GTs-protein interaction network. The GT-protein interaction network was composed of 151 GTs and 857 interacting proteins. (A) A network showing GTs and associated interacting proteins. Diamonds and round nodes represented GTs and interacting proteins, respectively. Node colors indicate the role of nodes in cancer and the role information was obtained from CancerMine database (http://bionlp.bcgsc.ca/cancermine/). (B) Top 10 GTs with the highest number of interacting proteins. GTs that had more interacting proteins included OGT, UGGT1, GYS1, STT3B, and DPM1. (C) GTs and interacting proteins that showed significantly positive correlations (correlation coefficient > 0.3). OGT was significantly positively correlated with the known tumor suppressor TET2 in 21 cancer types. UGGT1 was positively related to TP53BP1 in 20 cancer types. Besides, GALNT12 was positively associated with oncogenes MUC1 and PIK3CA in several cancer types. (D) GTs and interacting proteins that showed significantly negative correlations (correlation coefficient < -0.3). STT3B was found significantly negatively related to PTRH2 in 21 cancer types.

**Supplementary Figure 9. ﻿**FBXO6 was the most common interacted protein, especially associated with KDELC2 and higher expression of KDELC2 and FBXO6 collectively contributed to the poor prognosis in LGG. (A) FBXO6 was the most common interacted proteins. (B) FBXO6 was associated with the prognosis of patients in LGG, KIRC, and THYM. (C) The expression of FBXO6 was significantly associated with KDELC2 in LGG. (D) Higher expression of FBXO6 and KDELC2 collectively contributed to the poor prognosis in LGG.

**Supplementary Figure 10.** Functional annotations of GTs-related interacting proteins. (A) Functional enrichment analysis of the interacting proteins. Left panel, Gene ontology (GO) biological process enrichment. Right panel, KEGG pathway enrichment. (B) Proportions of interacting proteins with significant unfavorable or favorable prognostic effects (p < 0.05) across cancer types. The upper and lower bars, respectively, described results for GTs with unfavorable and favorable prognostic effects. For GTs that were identified as unfavorable prognosis factors, their corresponding interacting proteins tended to have the same prognostic impacts, especially in COAD, GBM, KIRP, LGG, LIHC, MESO, PAAD, STAD, and UVM. In contrast, in CESC, LUAD, MESO, and SKCM, for GTs that were identified as favorable prognosis factors, the proportions of interacting proteins which were also identified as favorable prognosis factors were higher.

**Supplementary Figure S11.** GTscore linked with the prognosis of patients and immunotherapy outcomes. (A) Distribution of GTscore across cancer types. (B) High GTscore group patients displayed poorer prognosis in 16 cancer types. (C) GTscore was associated with the prognosis of patients receiving PD1/PD-L1 immunotherapy. (D) The proportion of patients in the high GTscore group showed a lower response rate.

**Supplementary Figure S12.** Tumor microenvironment features between high and low GTscore groups.

**Supplementary Figure S13.** Comparison of the genetic and clinical characteristics between GTsCluster 1 and 2 in LIHC. (A) Somatic mutation features in GTsCluster 1. (B) Somatic mutation features in GTsCluster 2. (C) GTsCluster 1 exhibited lower TMB. (D) GTsCluster 1 exhibited a higher level of AFP. (E) The gender difference between GTsCluster 1 and 2 patients. (F) Histologic grade difference between GTsCluster 1 and 2 patients.

**Supplementary Figure S14.** Comparison of the CNV features between GTsCluster 1 and 2. (A) Focal-level copy number alterations. (B) Arm-level copy number alterations. GTsCluster 1 showed a higher burden of copy number gain at focal-level, while at both focal- and arm-level, GTsCluster 1 showed a higher burden of copy number loss.


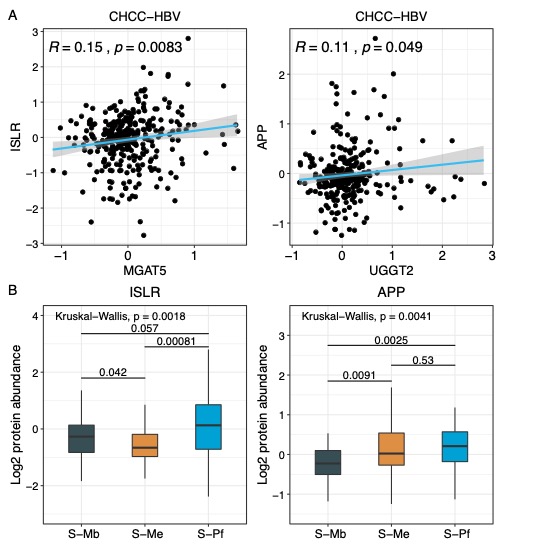


**Supplementary Figure 15. ﻿**The interacting proteins of MGAT5 and UGGT2 in LIHC. (A) The expression of MGAT5 was significantly correlated with ISLR, and the expression of UGGT2 was significantly correlated with APP. (B) Protein abundance of ISLR and APP in different subtypes of LIHC. ISLR and APP were upregulated in the proliferation subtype.

**Supplementary Figure S16.** Knockdown efficacy of GALNT4, MGAT5, and UGGT2 in LIHC cell lines (*: p < 0.05; **: p < 0.01; ***: p < 0.001).

**Supplementary Table S1.** GTs enrolled in this study.

**Supplementary Table S2.** Mutation frequency of GTs in TCGA and CCLE.

**Supplementary Table S3.** CNV frequency of GTs in TCGA and CCLE.

**Supplementary Table S4.** Differential gene expression analysis of GTs in TCGA.

**Supplementary Table S5.** Survival analysis of GTs.

**Supplementary Table S6.** SPIA analysis identifying GT-pathway interaction.

**Supplementary Table S7.** GT-protein interaction network.

**Supplementary Table S8.** Survival analysis of GTs in tumor samples with enriched CD8^+^ T cells.

**Supplementary Table S9.** GTs and assigned coefficients for GTscore calculation.
