# Supplementary figures and images for: An integrative pan‐cancer analysis of the molecular and biological features of glycosyltransferases
Source: Clin Transl Med. 2022 Jul 8;12(7):e872. doi: 10.1002/ctm2.872 (PMC9270580; doi:10.1002/ctm2.872)

A

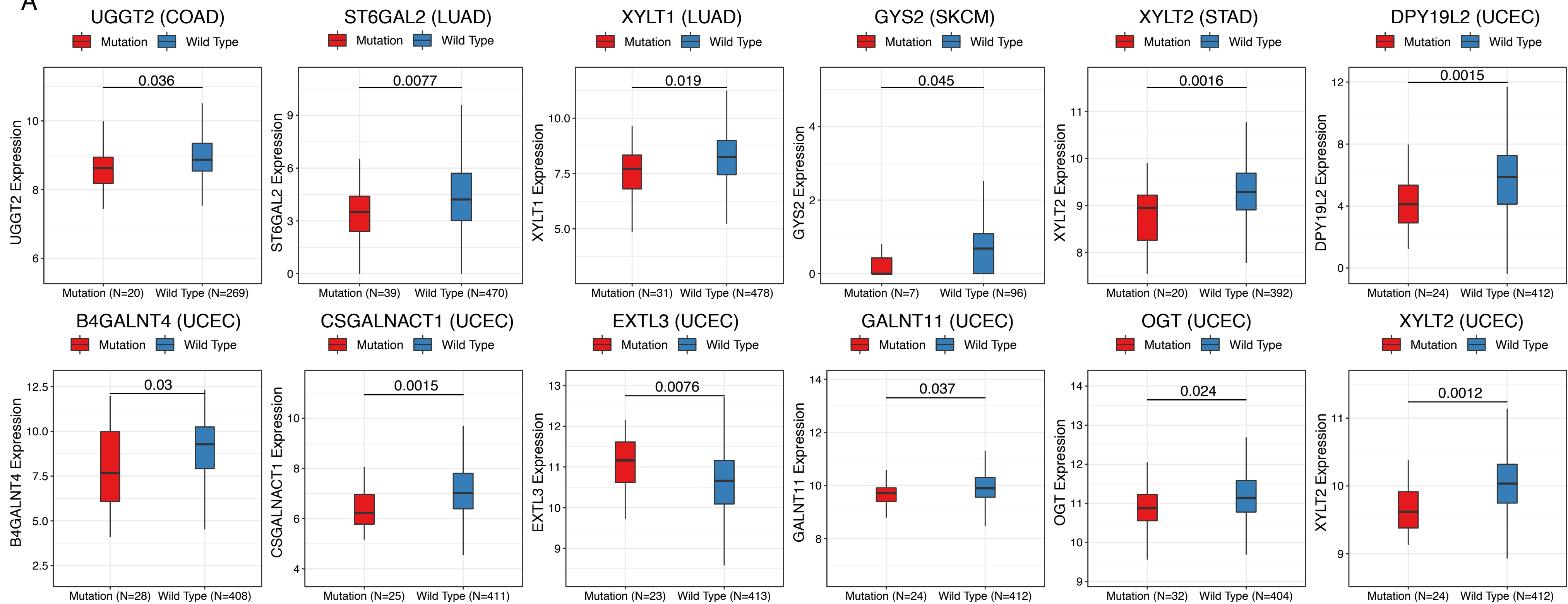

B

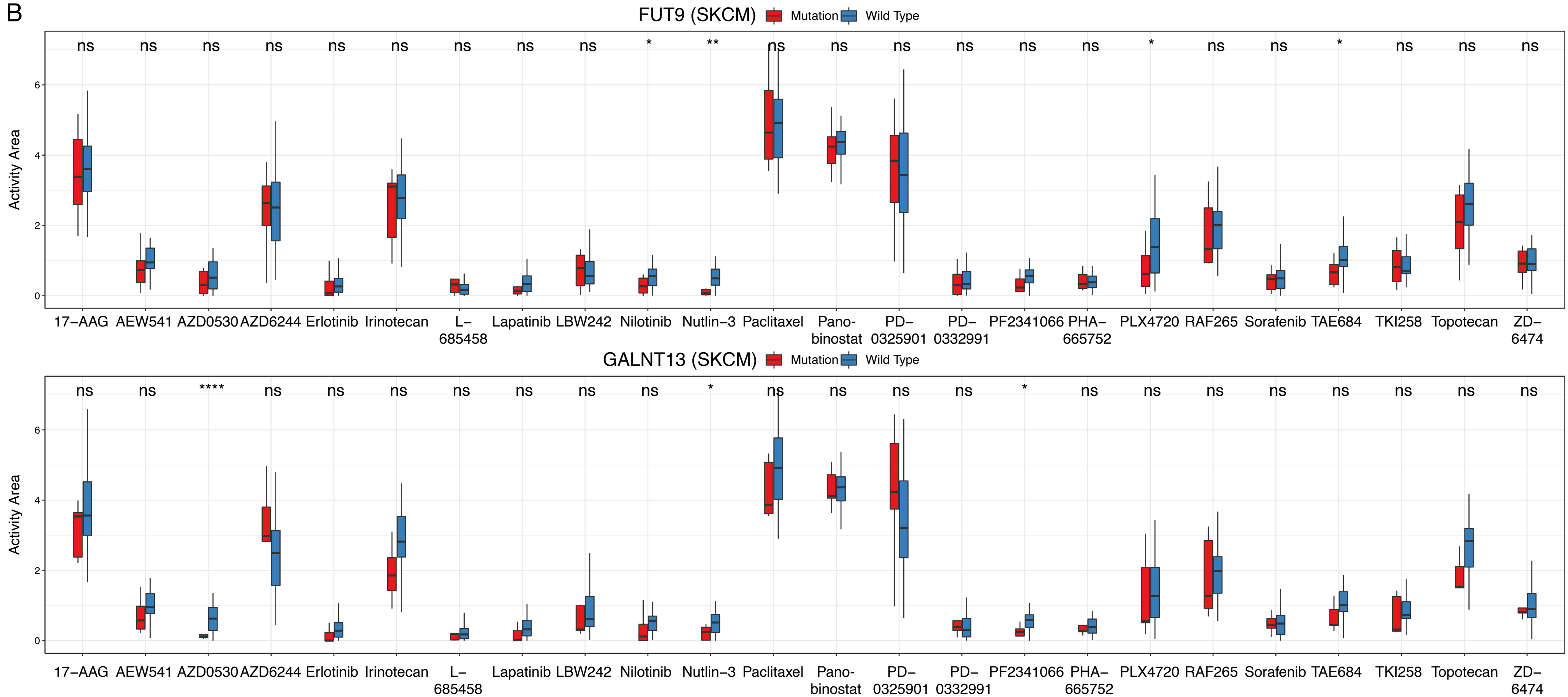

Supplement: Supplementary file 1 — Supporting Information [file CTM2-12-e872-s001.zip › ctm2872-sup-0001-SuppMat/SupplementaryFiles20220516/SFigure/S1.pdf]

A

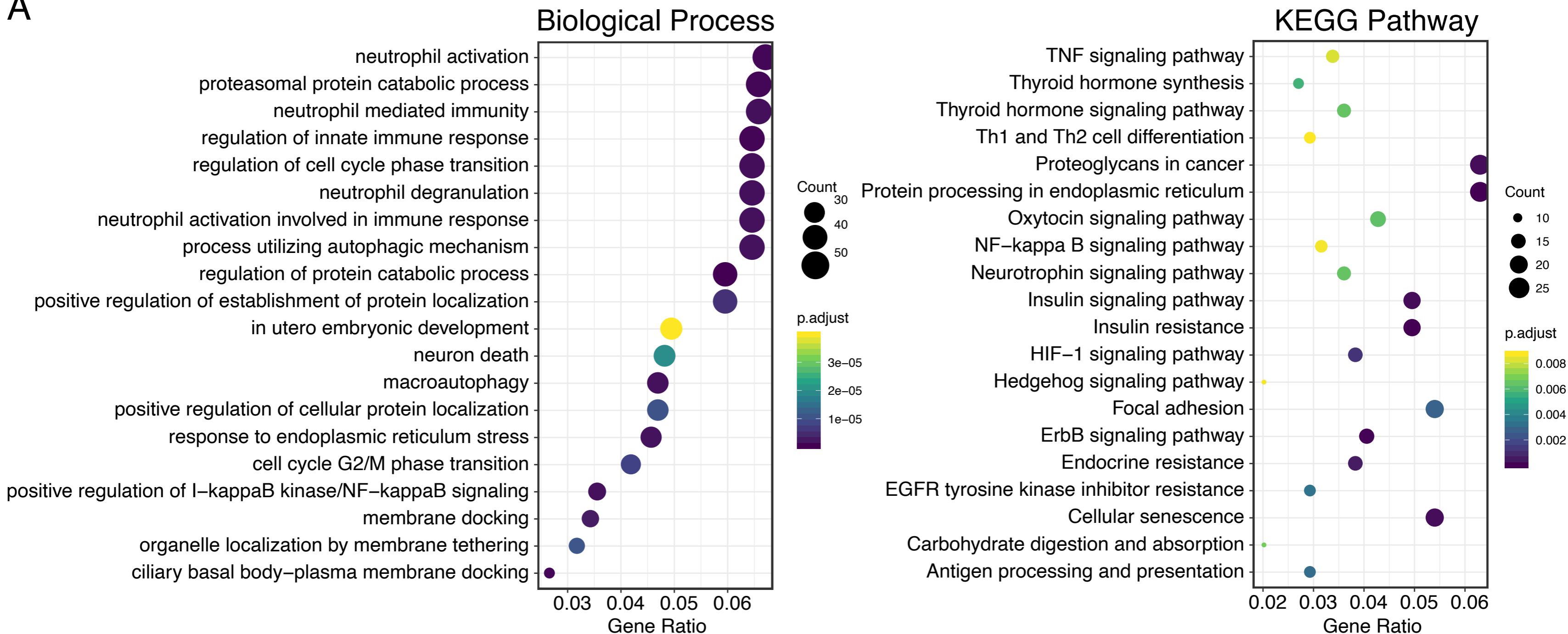

B

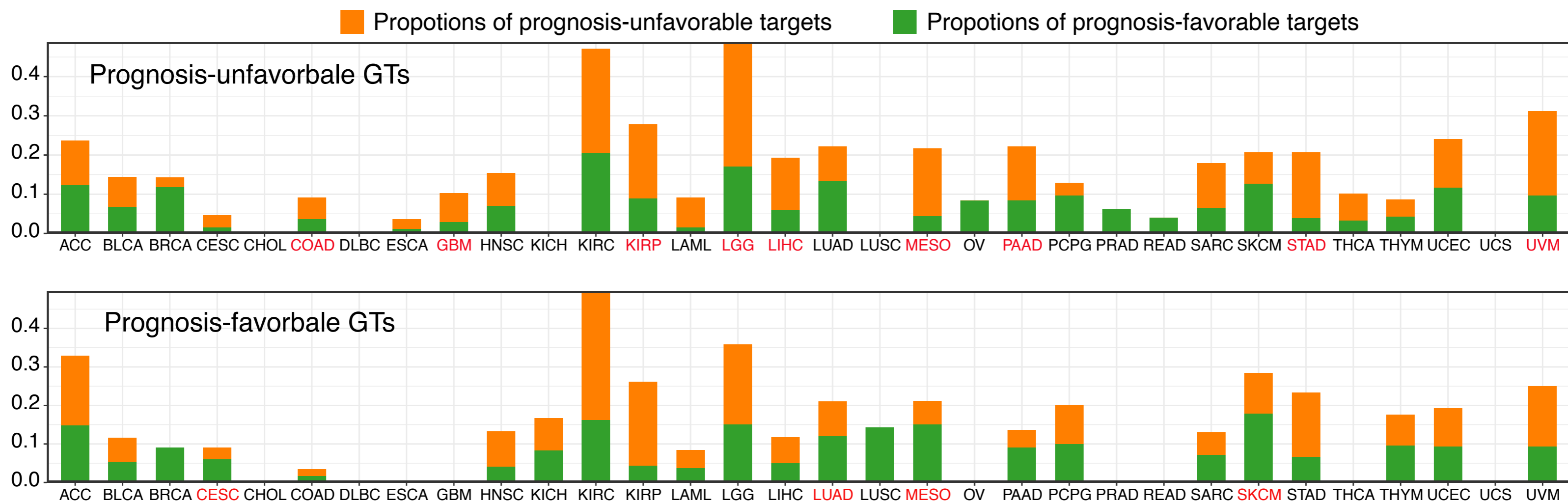

Supplement: Supplementary file 1 — Supporting Information [file CTM2-12-e872-s001.zip › ctm2872-sup-0001-SuppMat/SupplementaryFiles20220516/SFigure/S10.pdf]

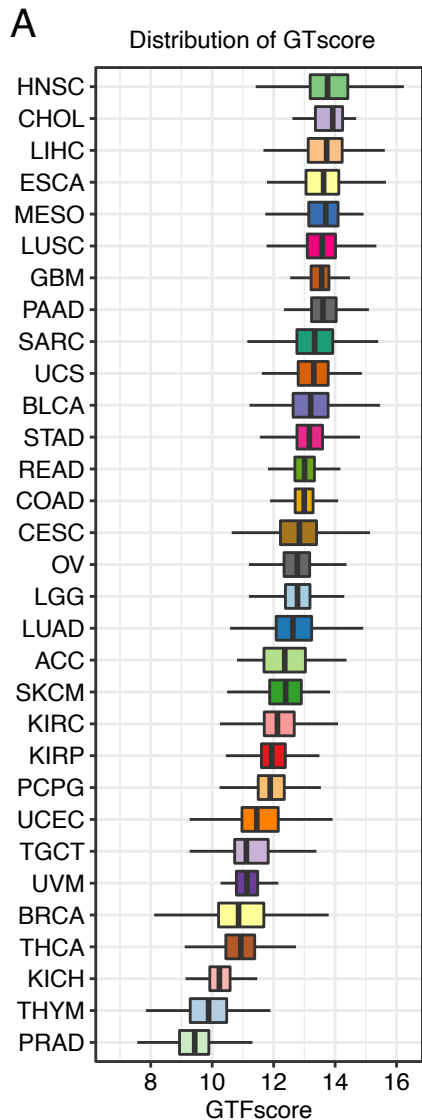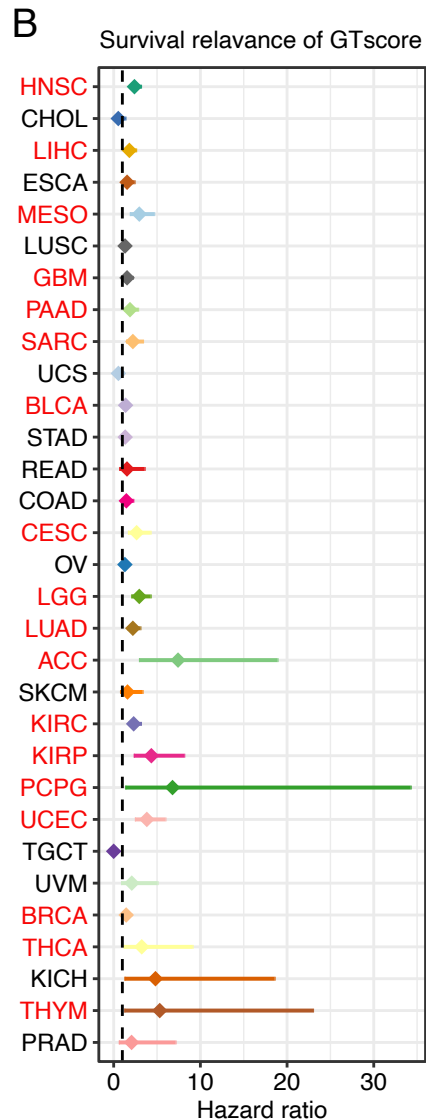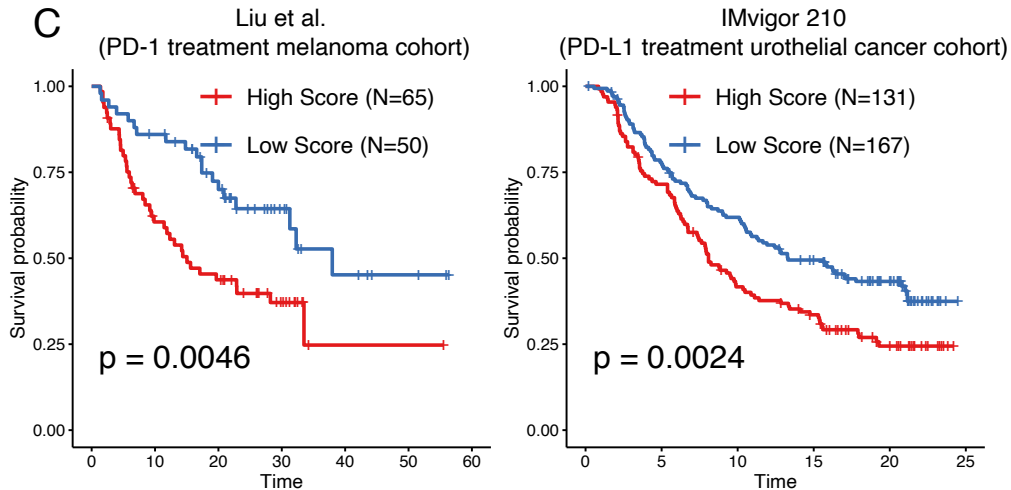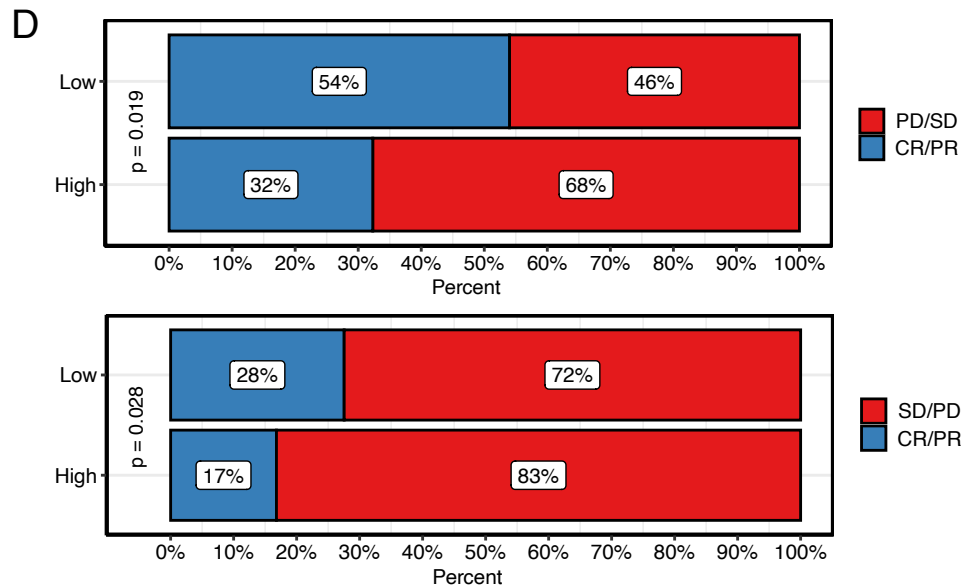

Supplement: Supplementary file 1 — Supporting Information [file CTM2-12-e872-s001.zip › ctm2872-sup-0001-SuppMat/SupplementaryFiles20220516/SFigure/S11.pdf]

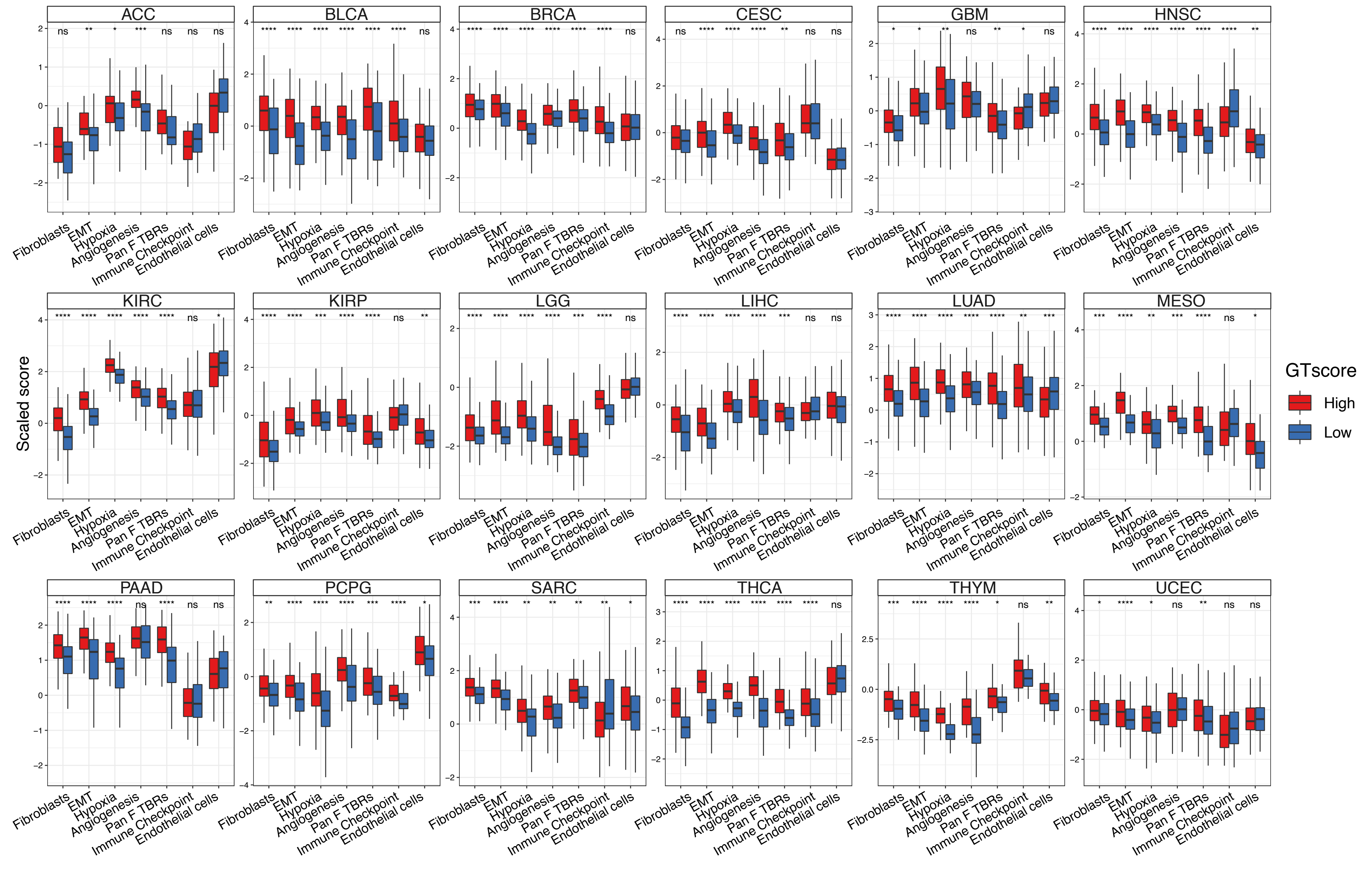

Supplement: Supplementary file 1 — Supporting Information [file CTM2-12-e872-s001.zip › ctm2872-sup-0001-SuppMat/SupplementaryFiles20220516/SFigure/S12.pdf]

**A** Altered in 91 (88.35%) of 103 samples.

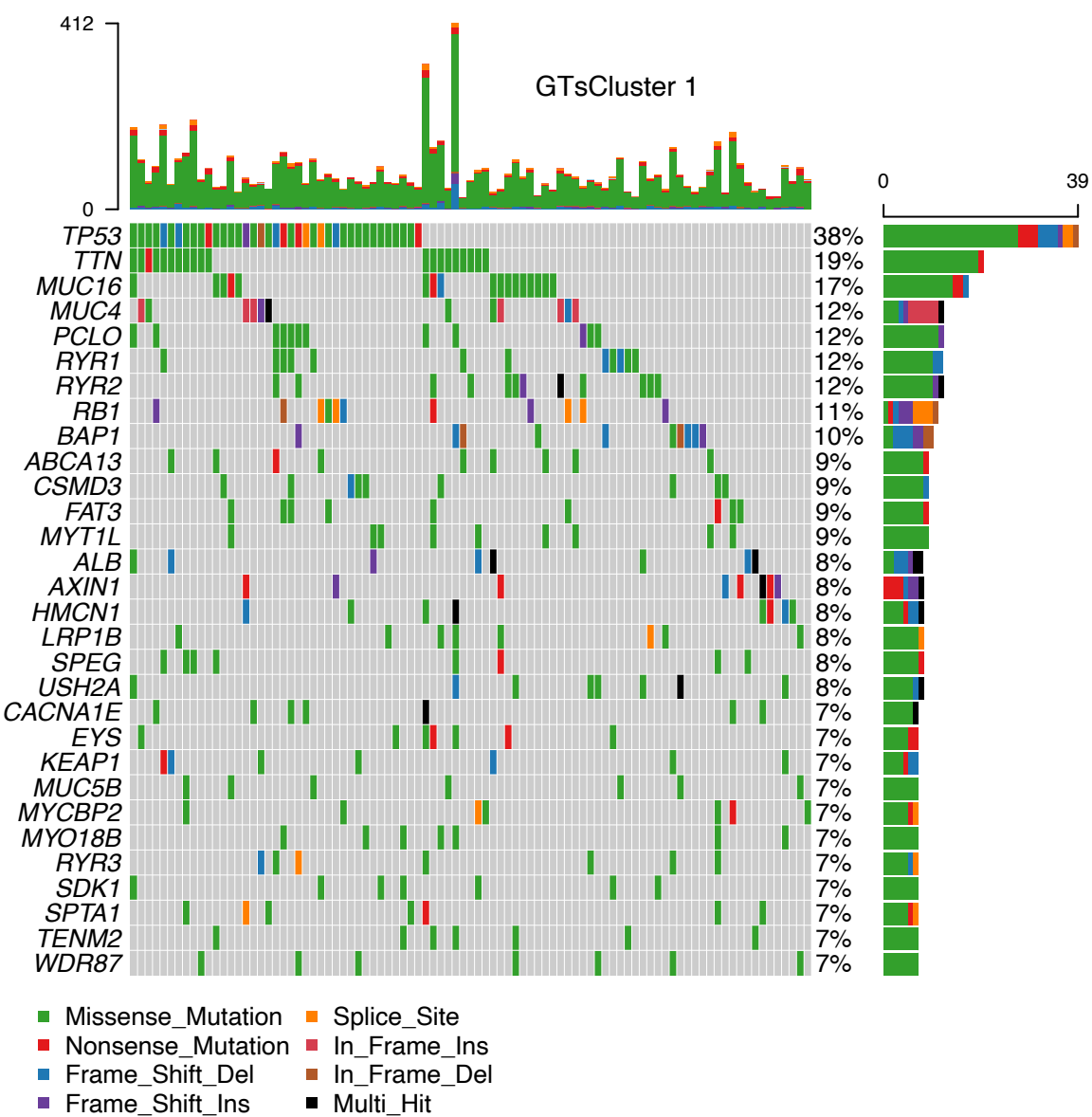

**B** Altered in 211 (92.54%) of 228 samples.

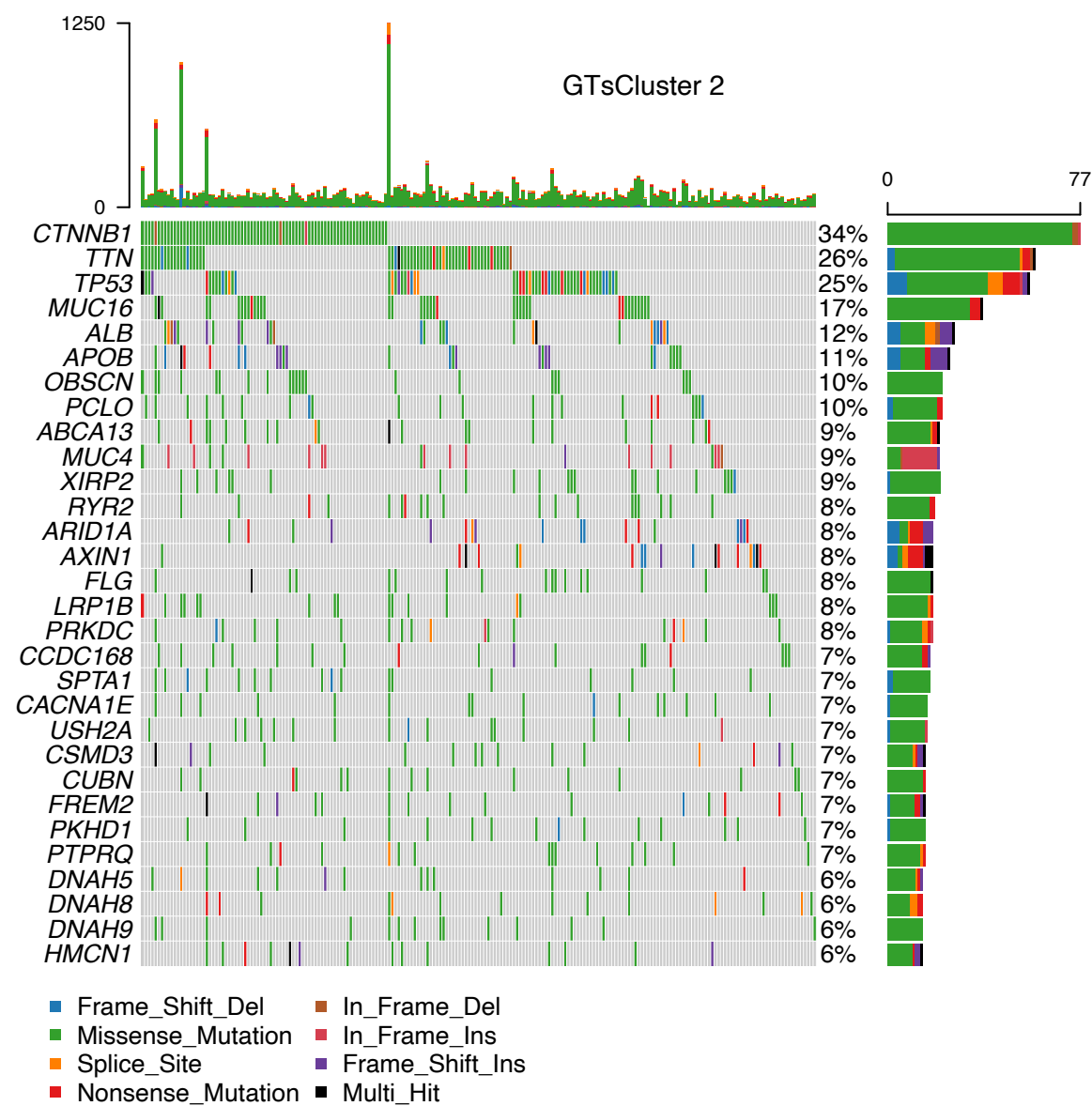

**C**

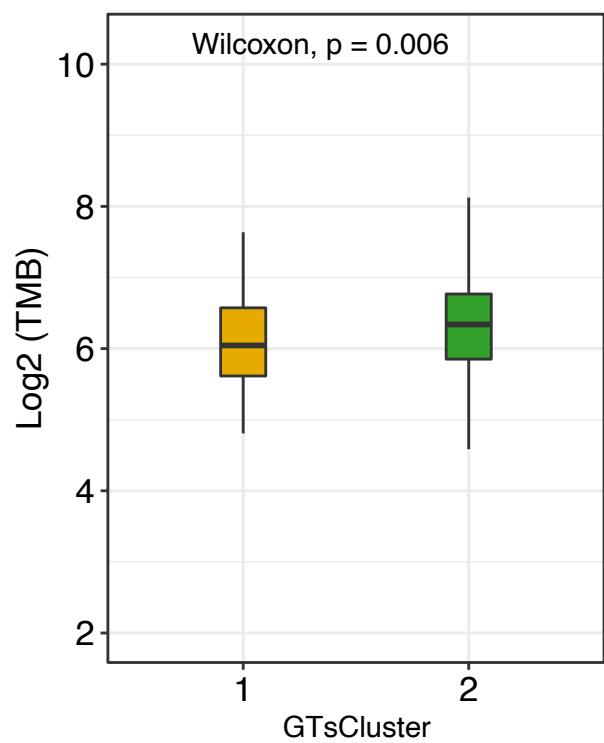

**D**

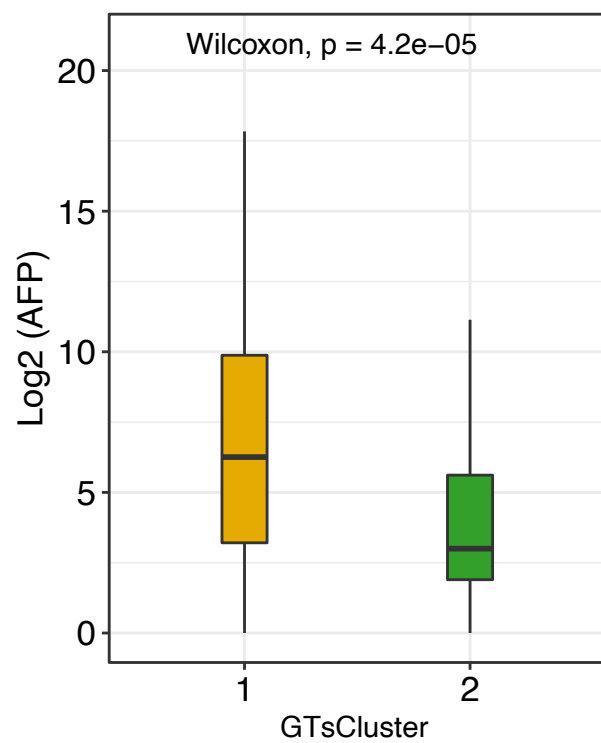

**E**

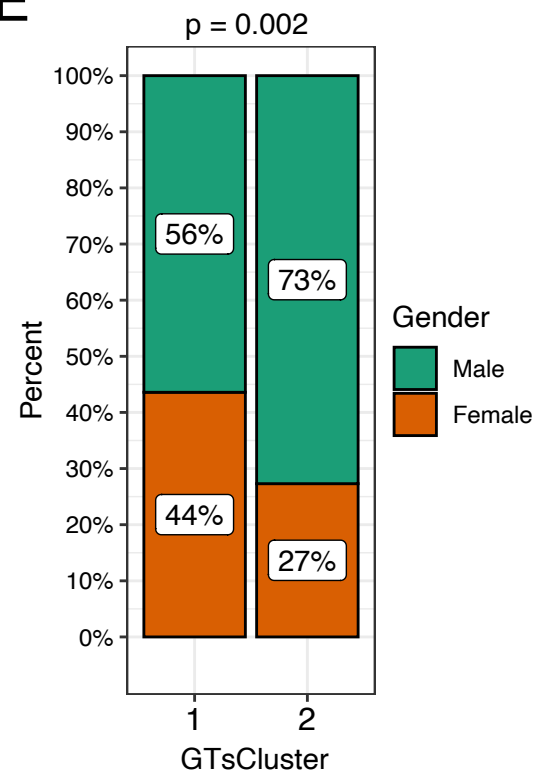

**F**

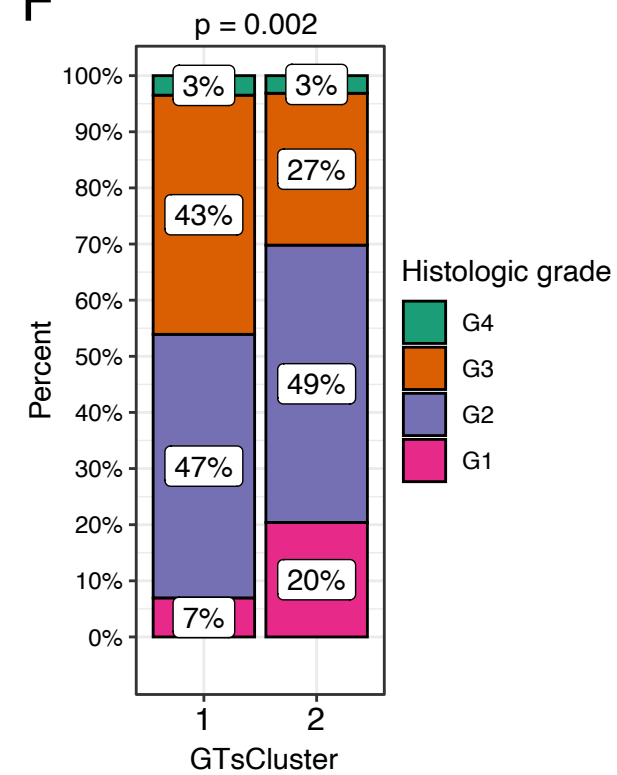

Supplement: Supplementary file 1 — Supporting Information [file CTM2-12-e872-s001.zip › ctm2872-sup-0001-SuppMat/SupplementaryFiles20220516/SFigure/S13.pdf]

A

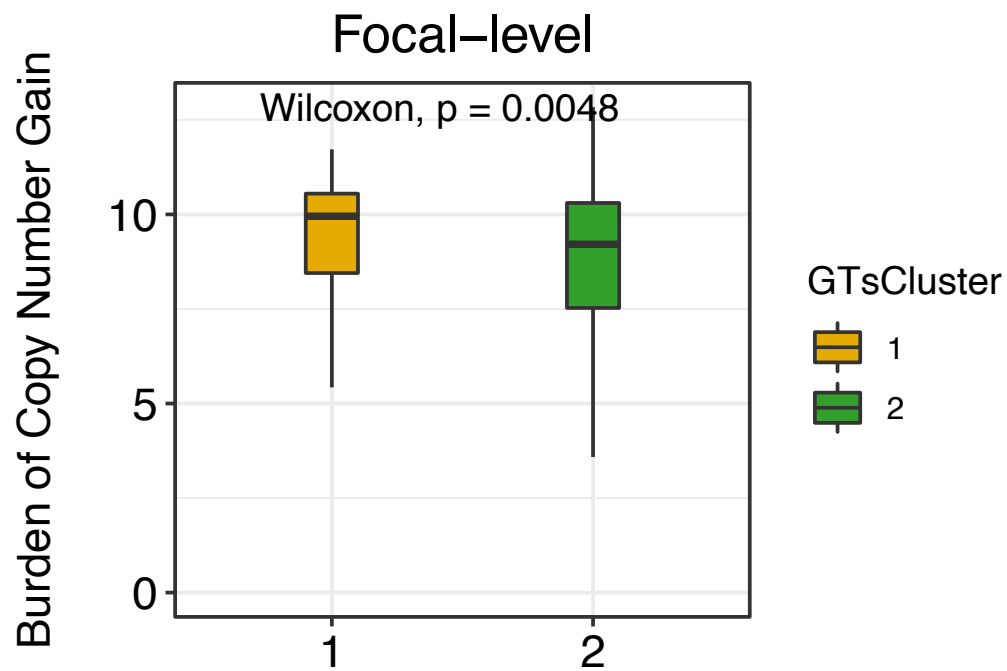

B

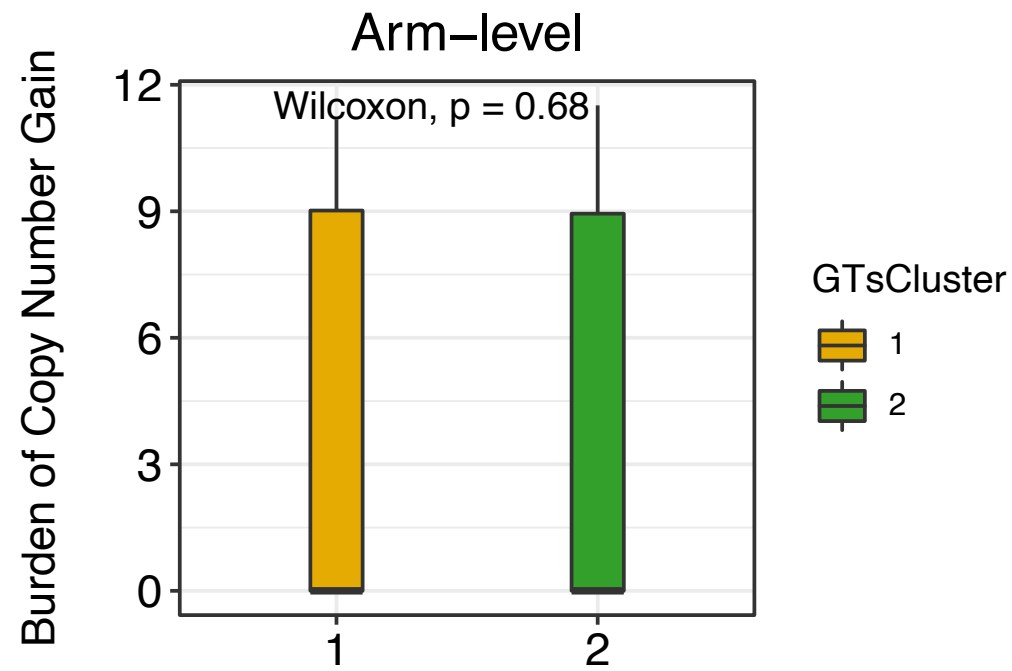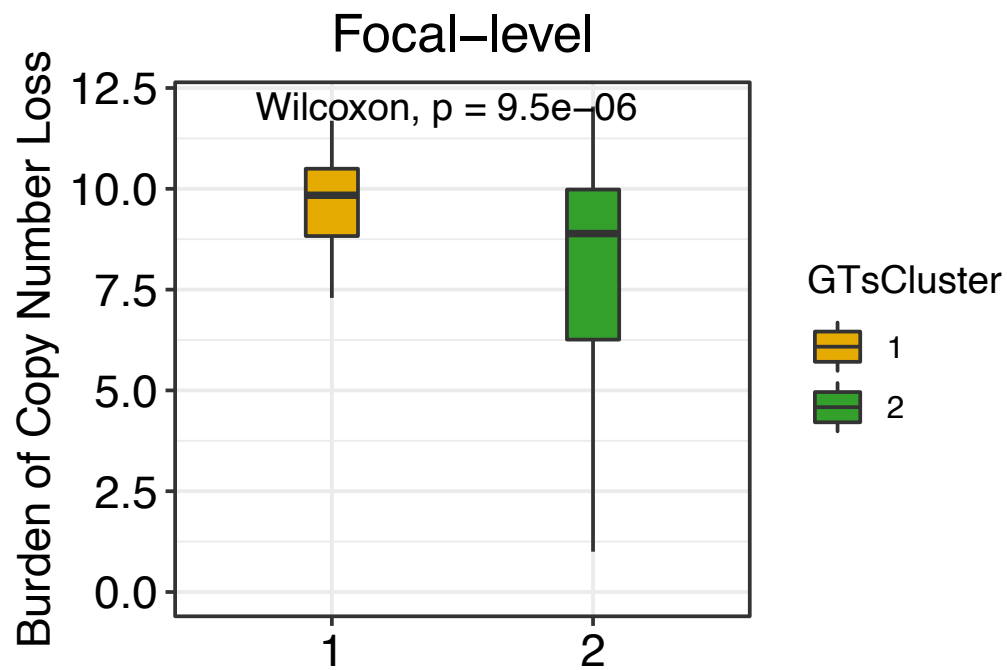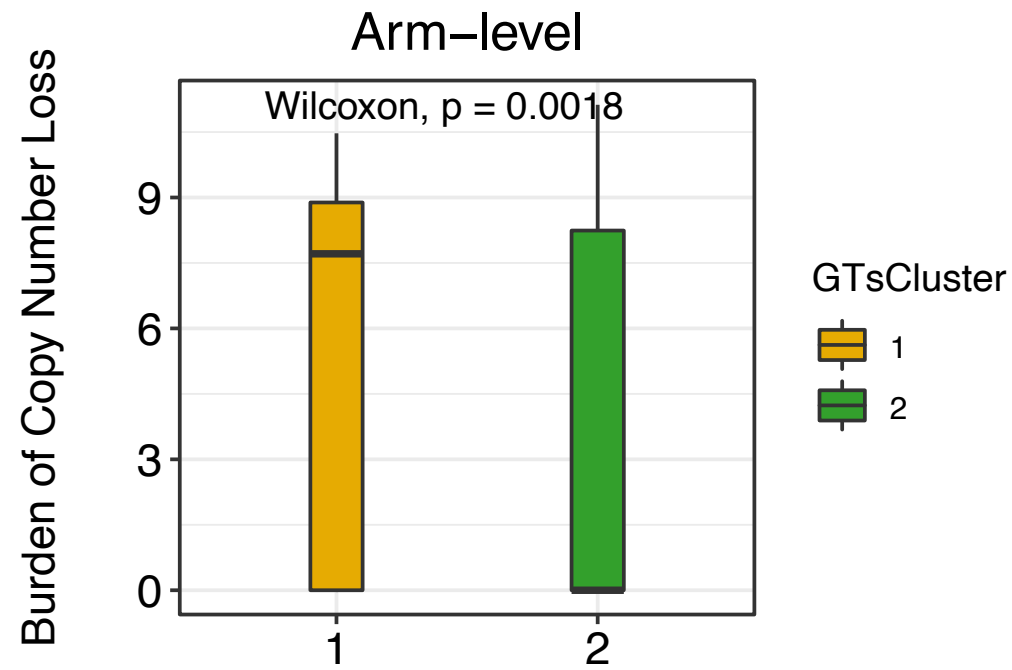

Supplement: Supplementary file 1 — Supporting Information [file CTM2-12-e872-s001.zip › ctm2872-sup-0001-SuppMat/SupplementaryFiles20220516/SFigure/S14.pdf]

A

CHCC-HBV

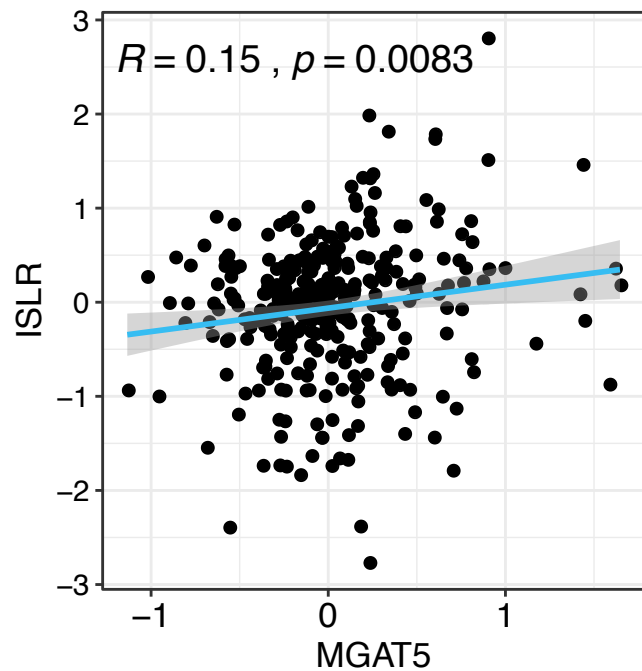

CHCC-HBV

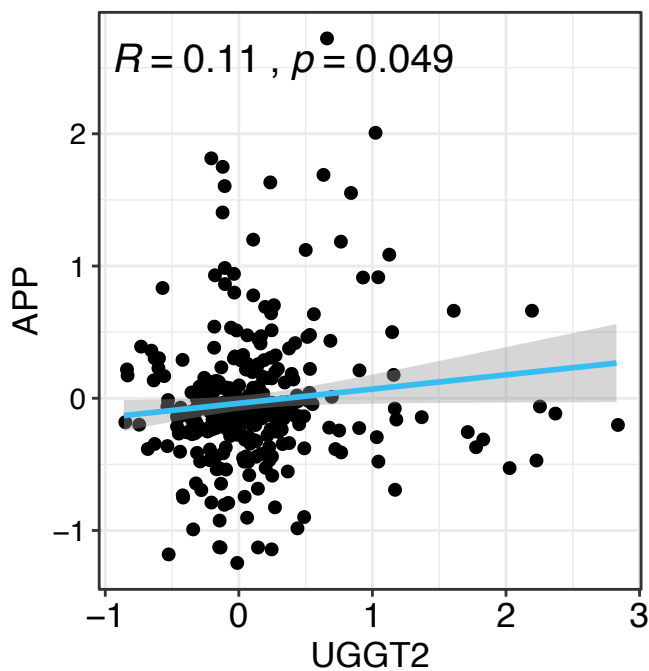

B

ISLR

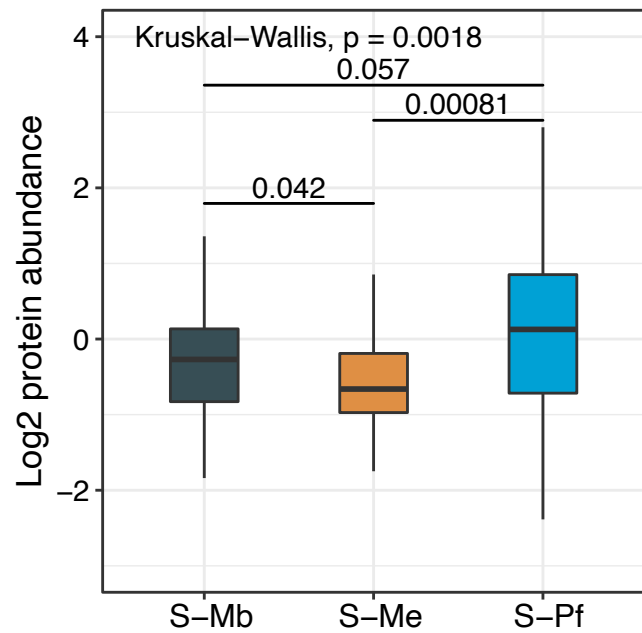

APP

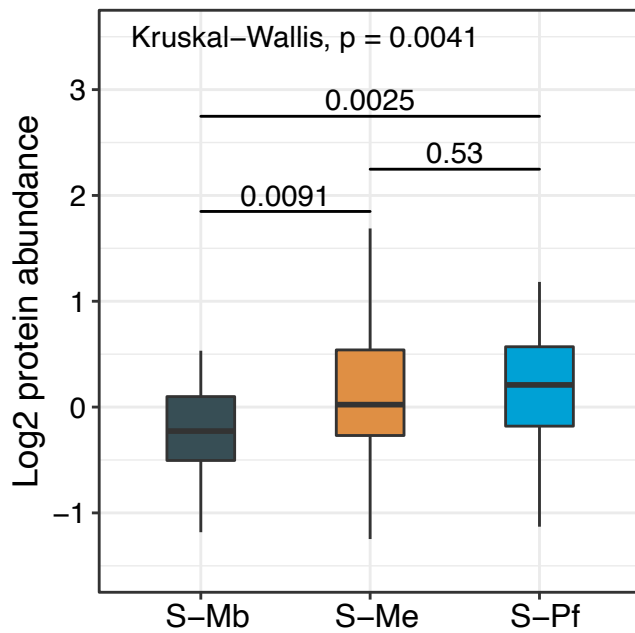

Supplement: Supplementary file 1 — Supporting Information [file CTM2-12-e872-s001.zip › ctm2872-sup-0001-SuppMat/SupplementaryFiles20220516/SFigure/S15.pdf]

PLC

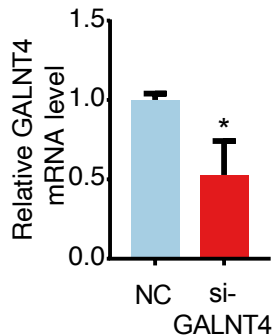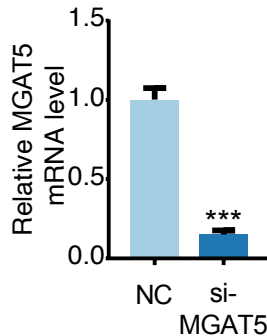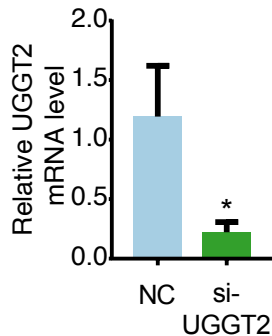

HepG2

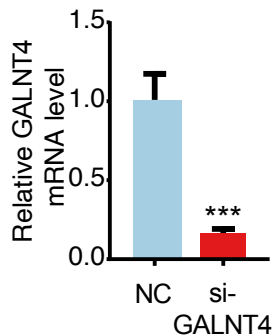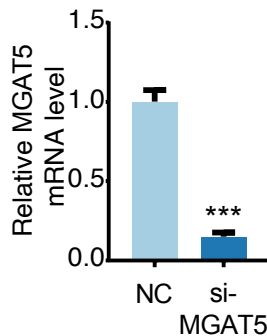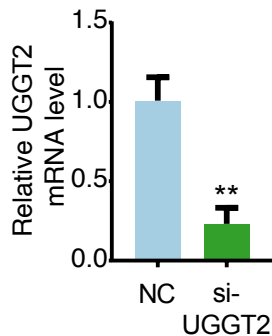

Supplement: Supplementary file 1 — Supporting Information [file CTM2-12-e872-s001.zip › ctm2872-sup-0001-SuppMat/SupplementaryFiles20220516/SFigure/S16.pdf]

A

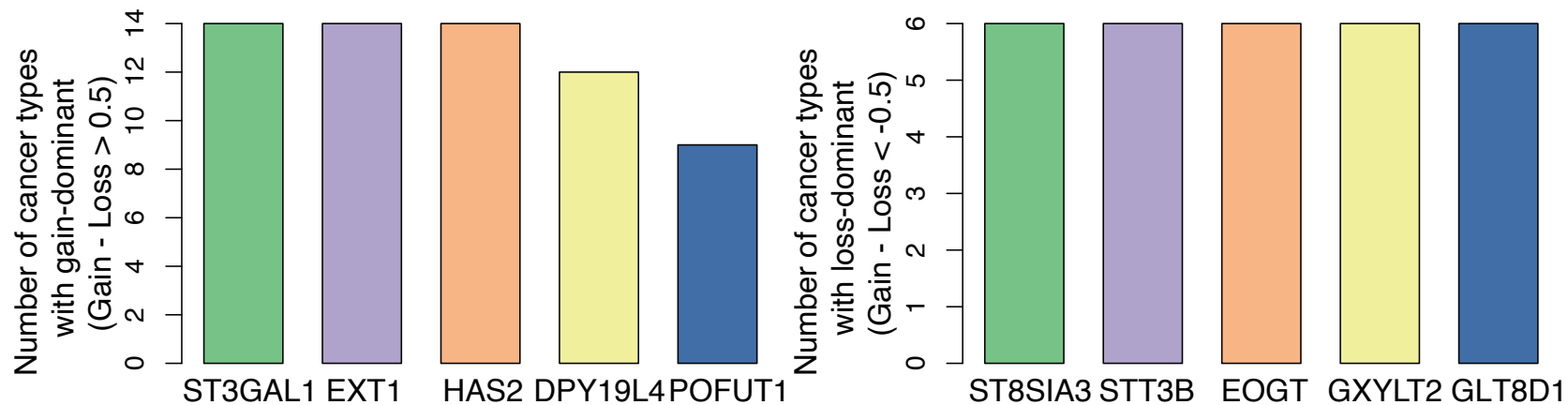

B

Correlation with gene expression ( $p < 0.05$ )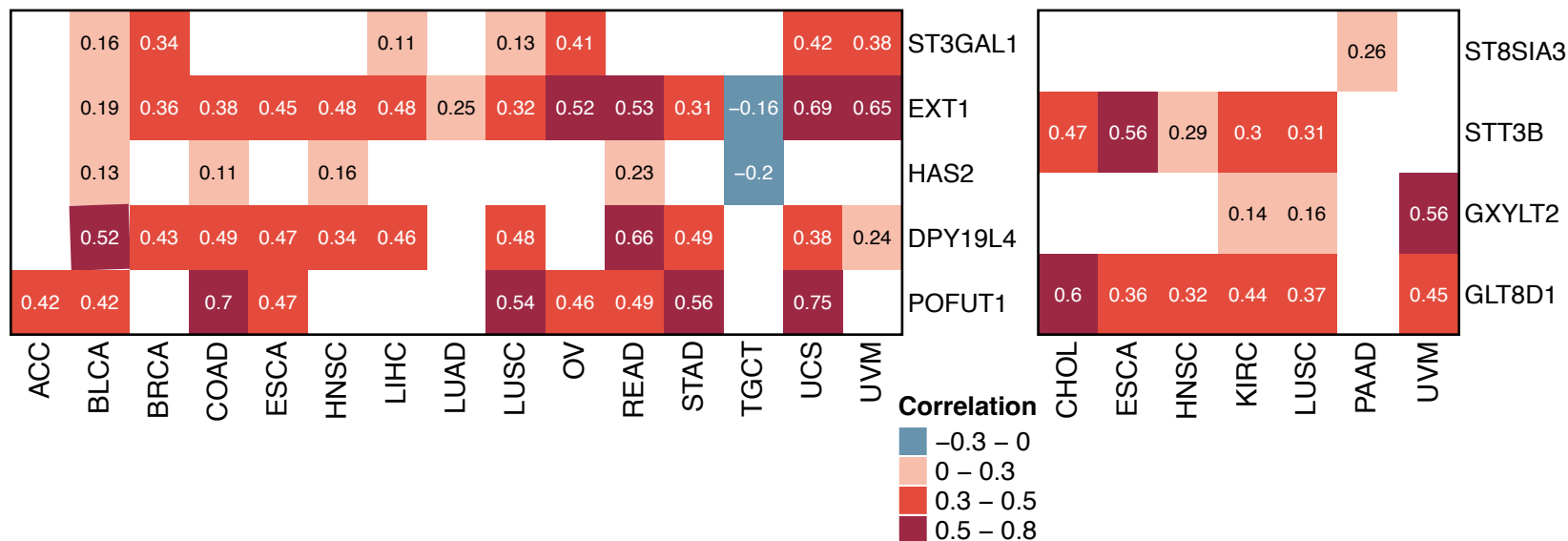

Supplement: Supplementary file 1 — Supporting Information [file CTM2-12-e872-s001.zip › ctm2872-sup-0001-SuppMat/SupplementaryFiles20220516/SFigure/S2.pdf]

A

CCLE Mutation frequency

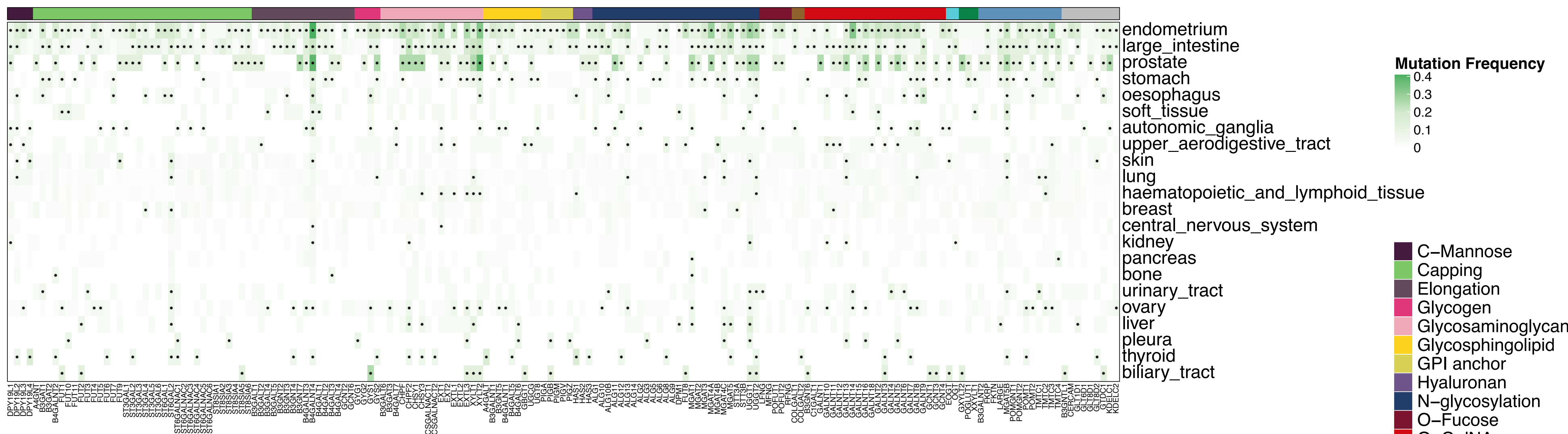

B

CCLE CNV frequency

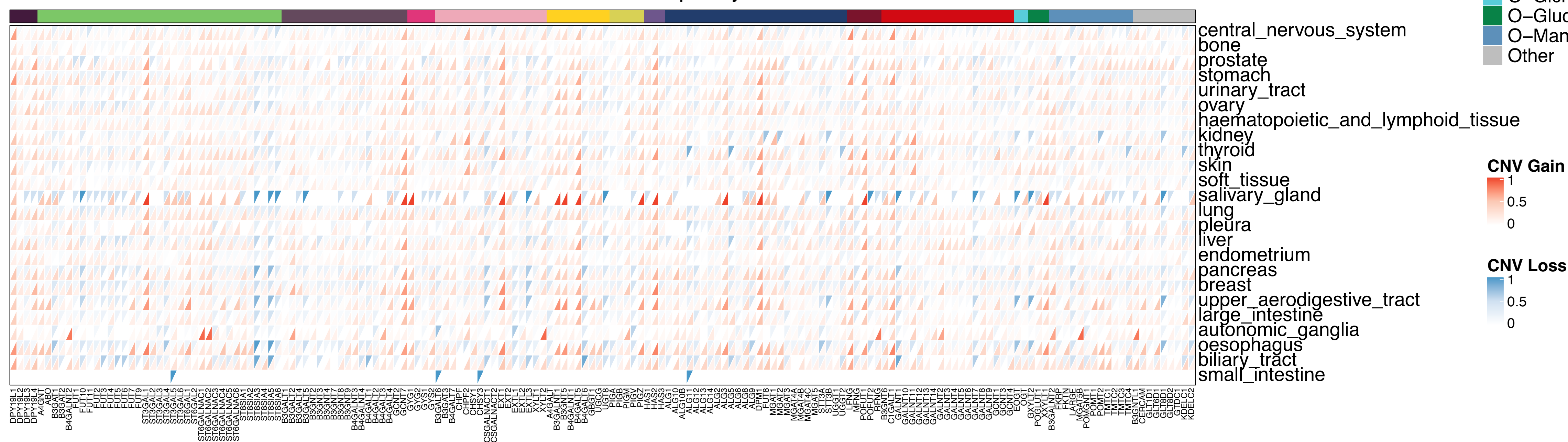

C

TCGA

CCLE

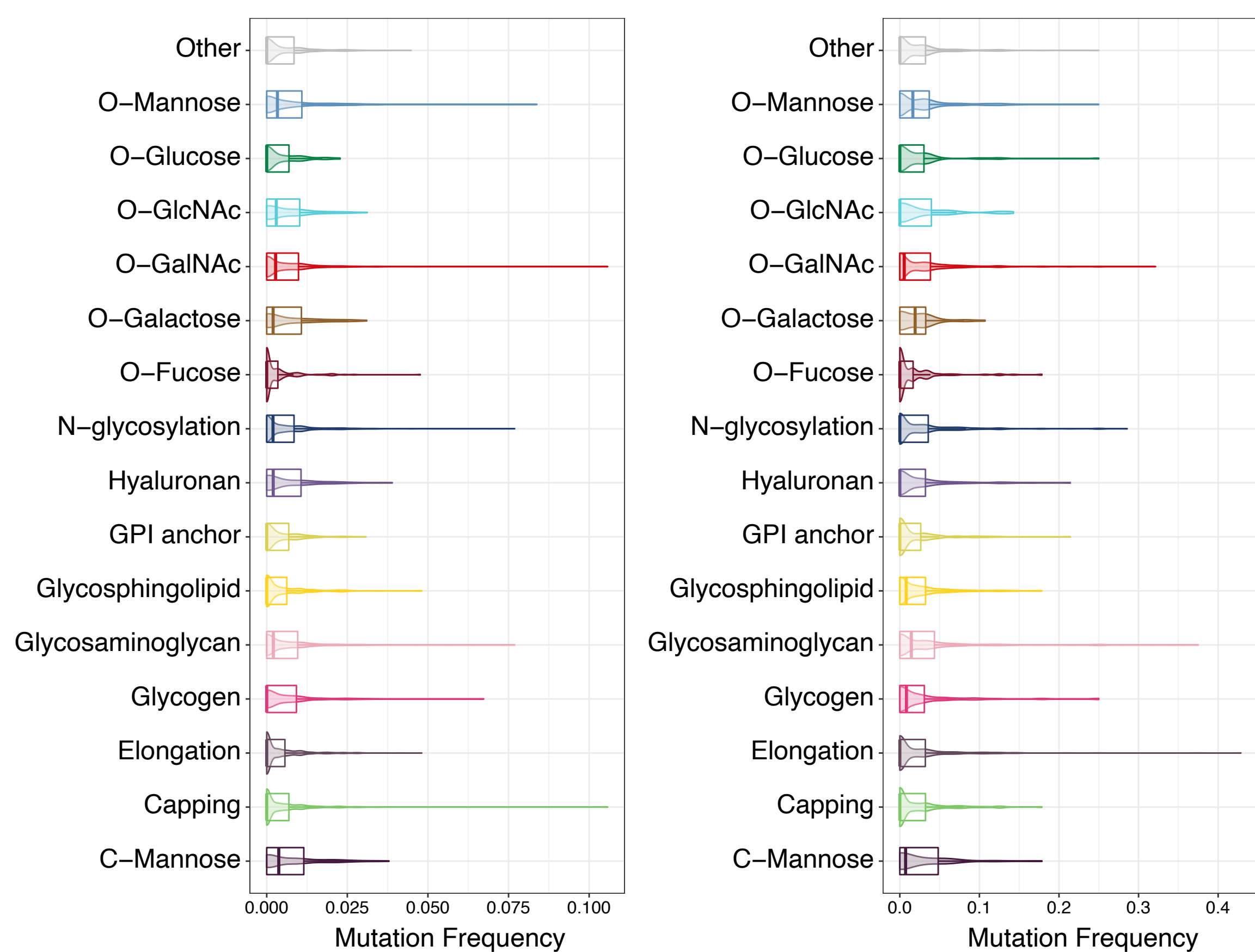

D

TCGA

CCLE

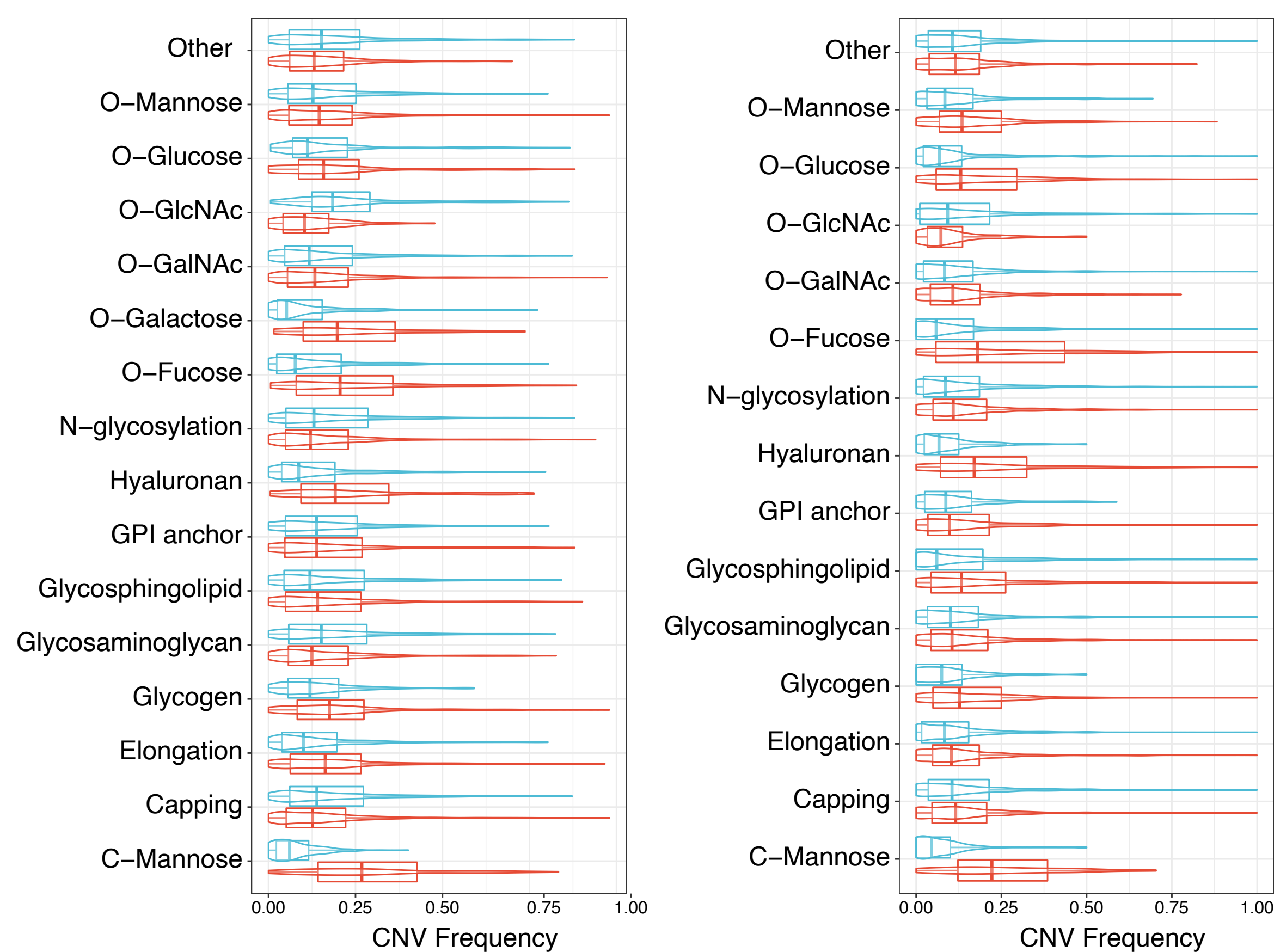

Supplement: Supplementary file 1 — Supporting Information [file CTM2-12-e872-s001.zip › ctm2872-sup-0001-SuppMat/SupplementaryFiles20220516/SFigure/S3.pdf]

A

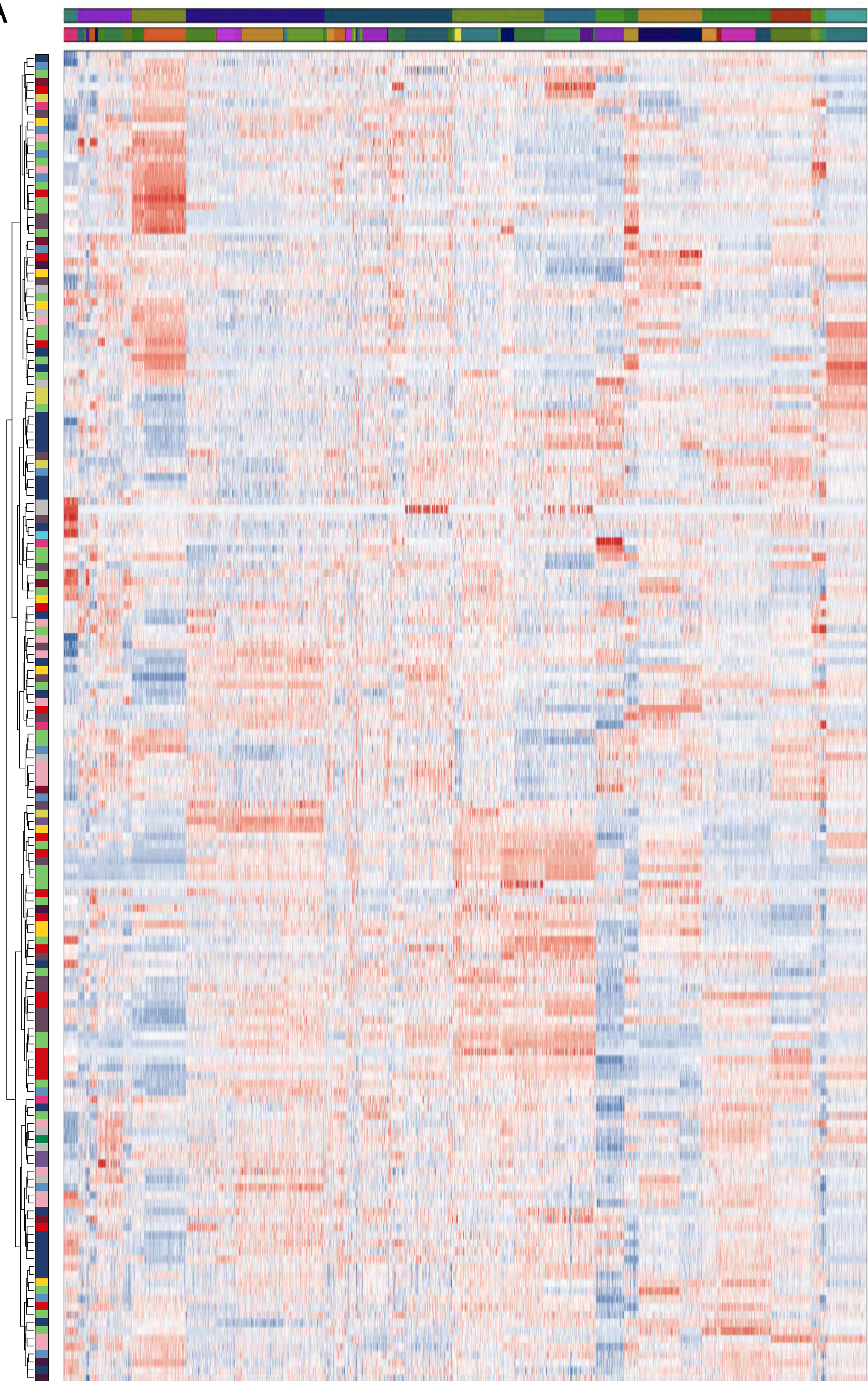

B

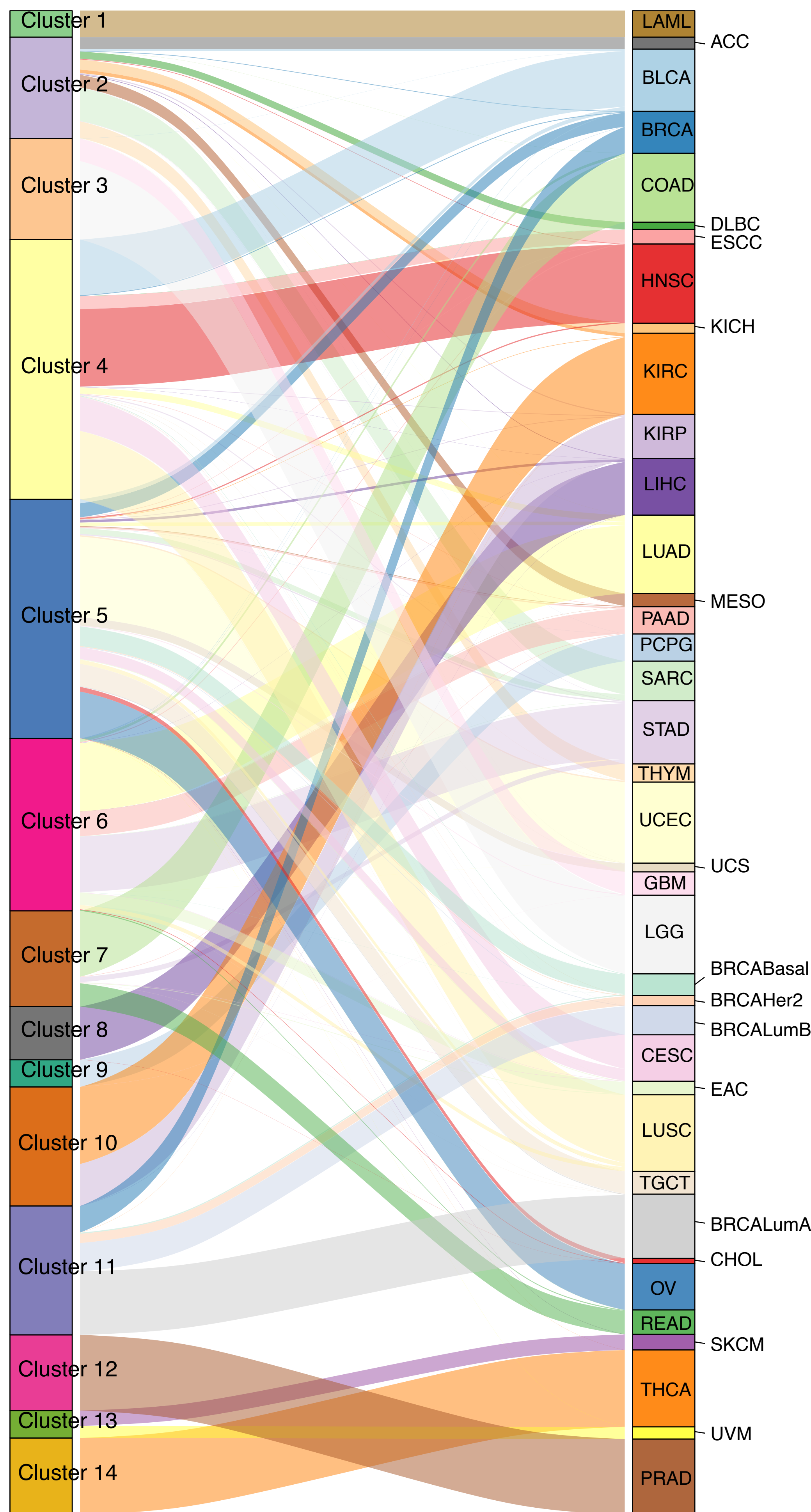

Supplement: Supplementary file 1 — Supporting Information [file CTM2-12-e872-s001.zip › ctm2872-sup-0001-SuppMat/SupplementaryFiles20220516/SFigure/S4.pdf]

A

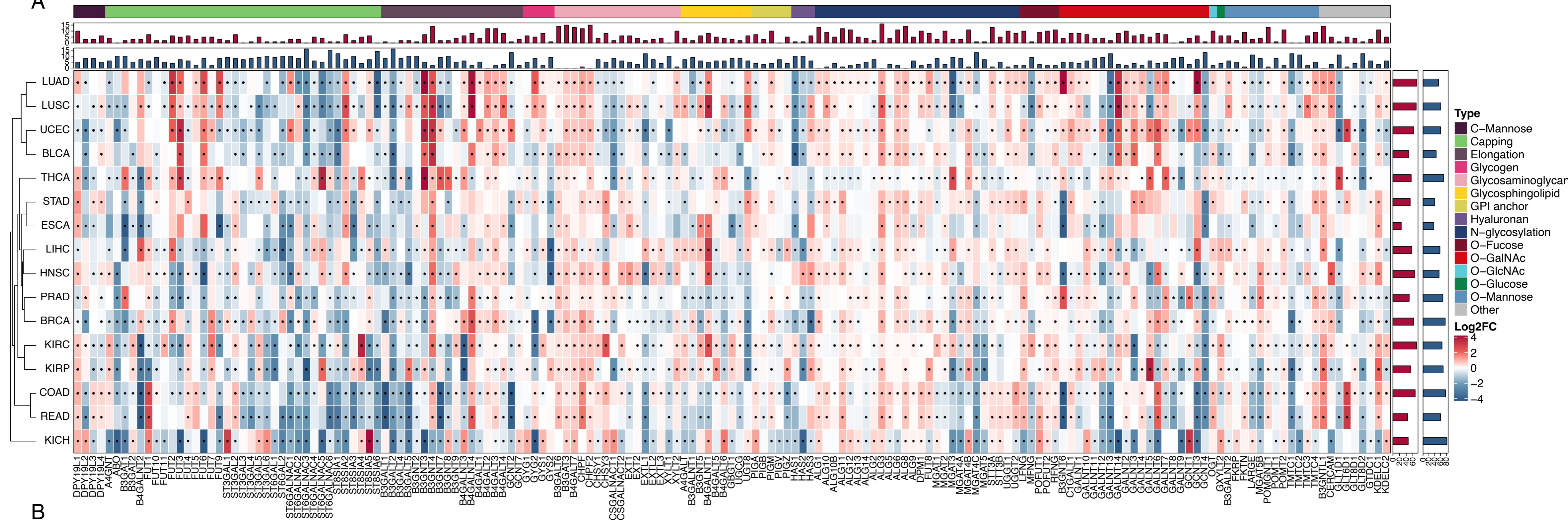

B

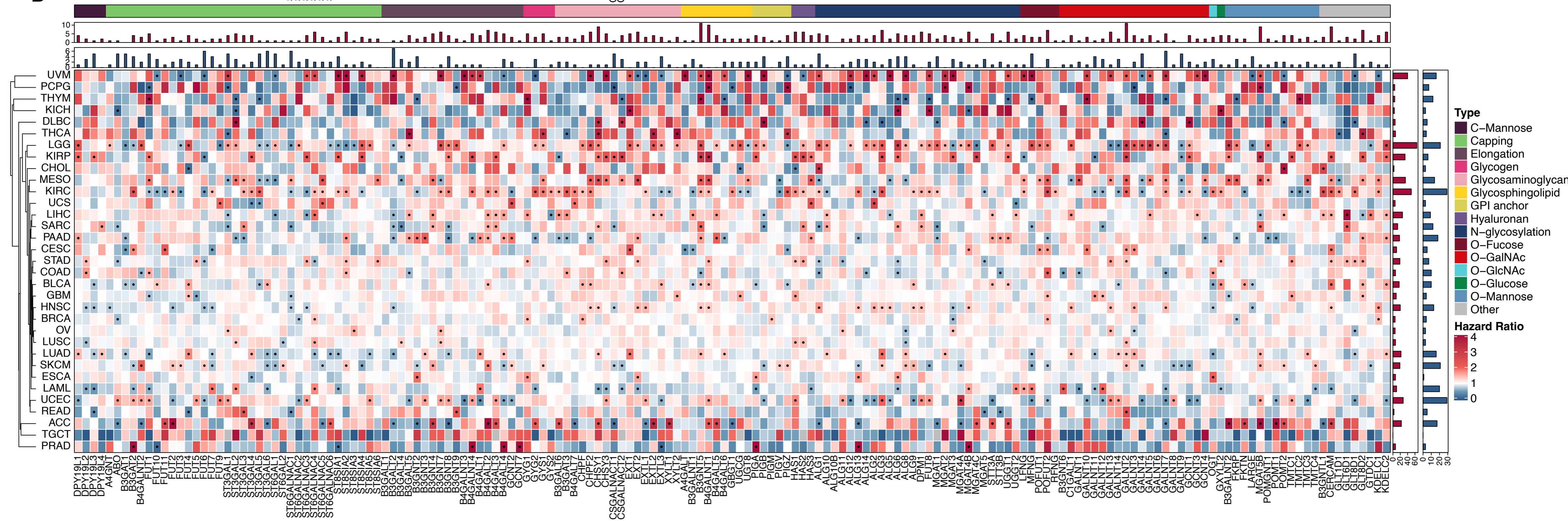

Supplement: Supplementary file 1 — Supporting Information [file CTM2-12-e872-s001.zip › ctm2872-sup-0001-SuppMat/SupplementaryFiles20220516/SFigure/S5.pdf]

A

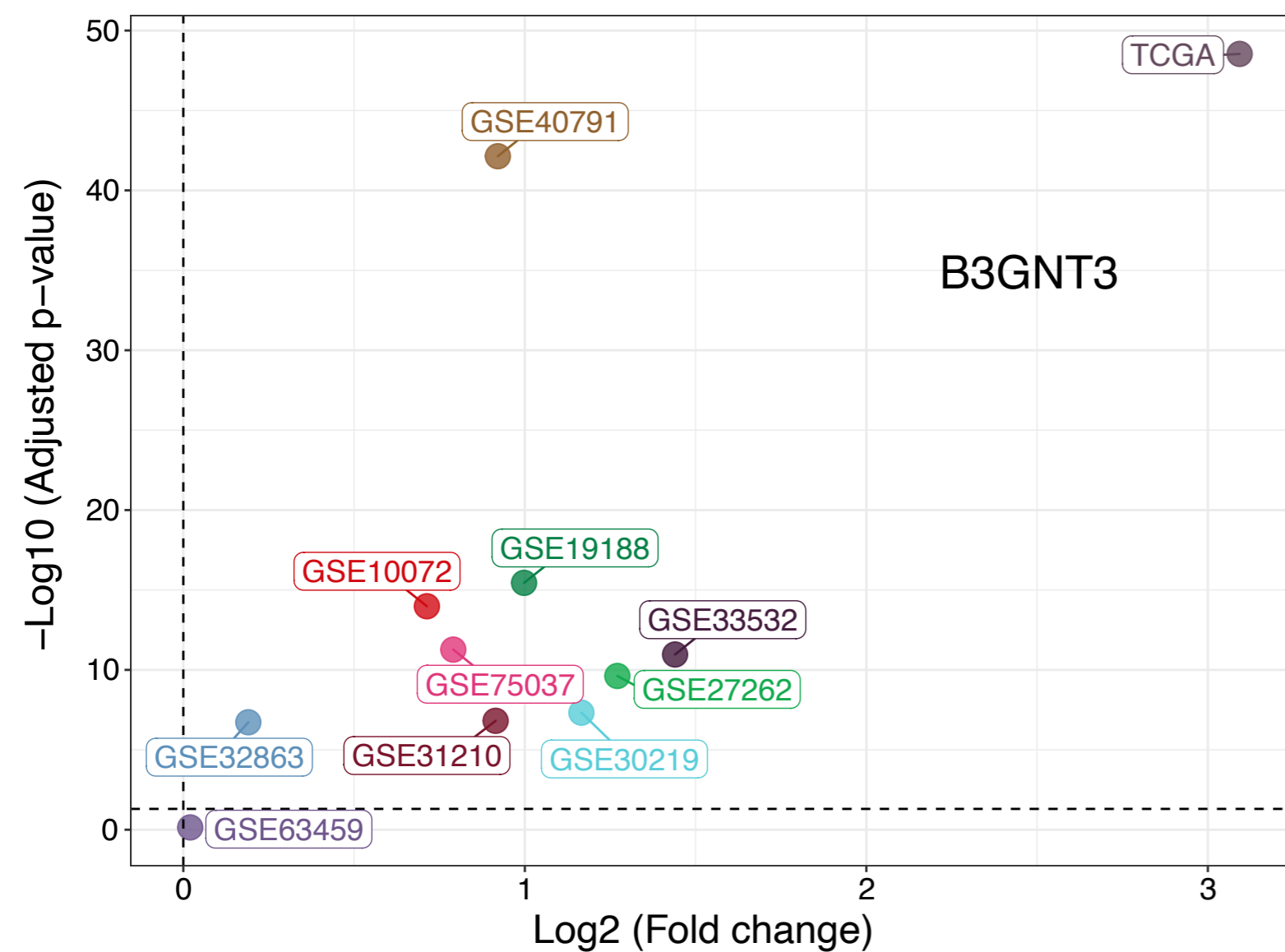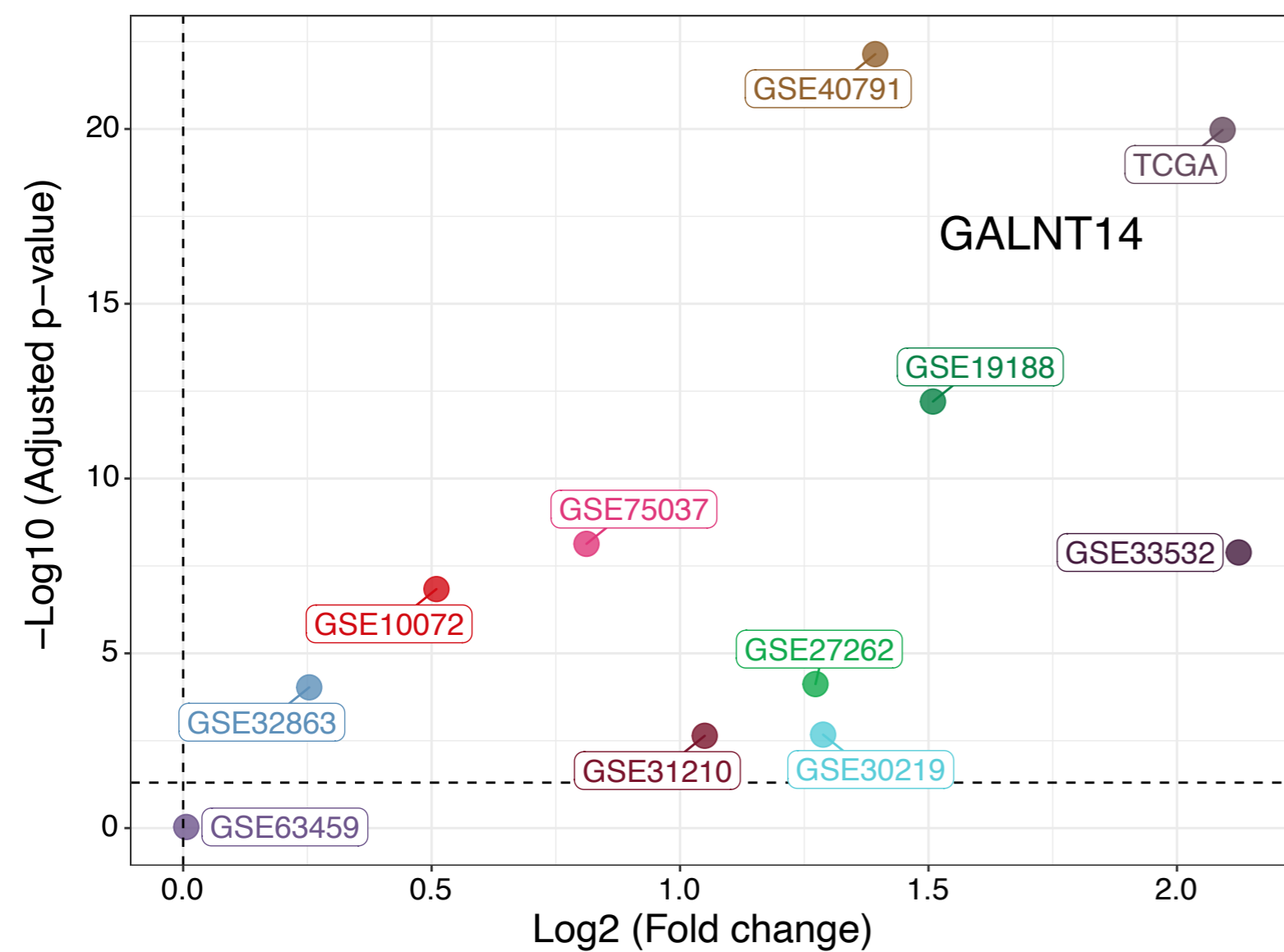

B

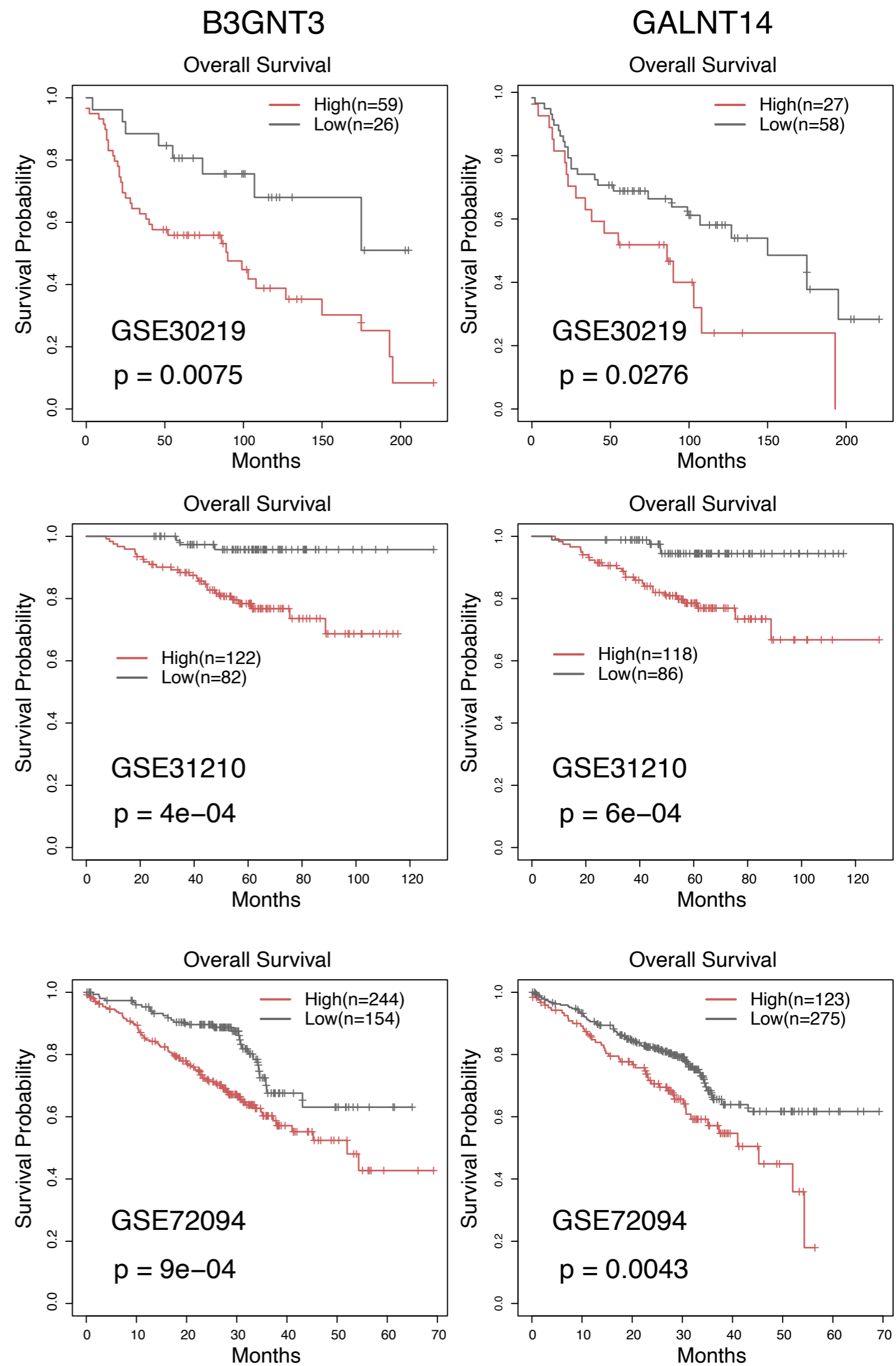

Supplement: Supplementary file 1 — Supporting Information [file CTM2-12-e872-s001.zip › ctm2872-sup-0001-SuppMat/SupplementaryFiles20220516/SFigure/S6.pdf]

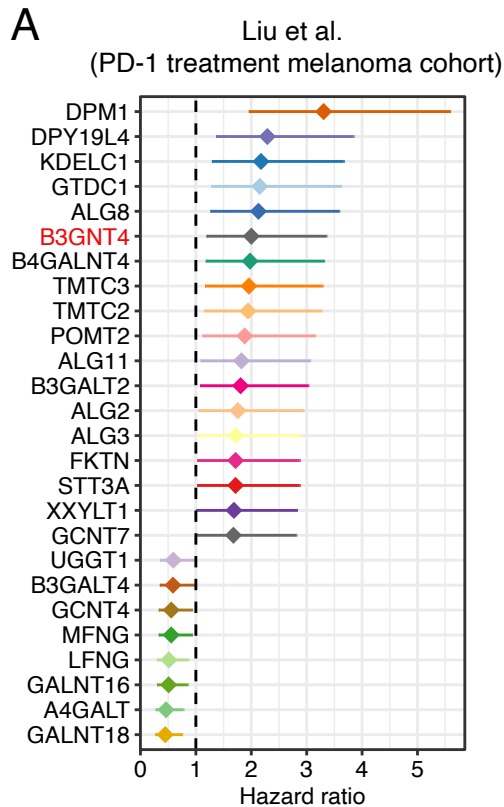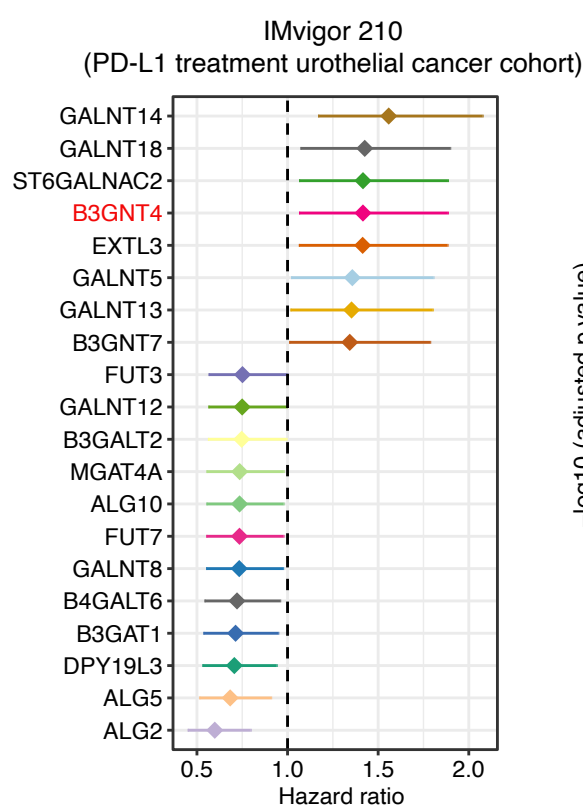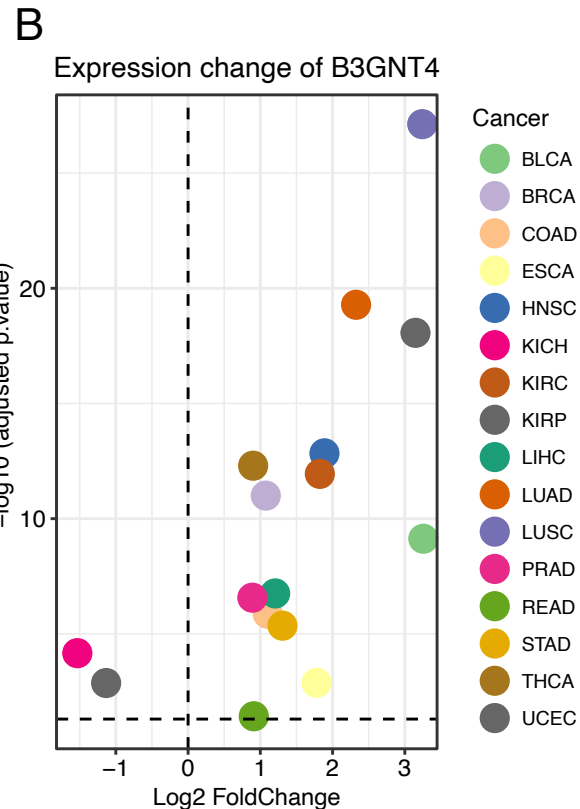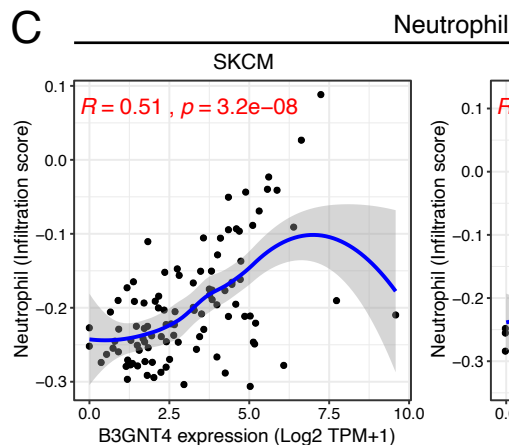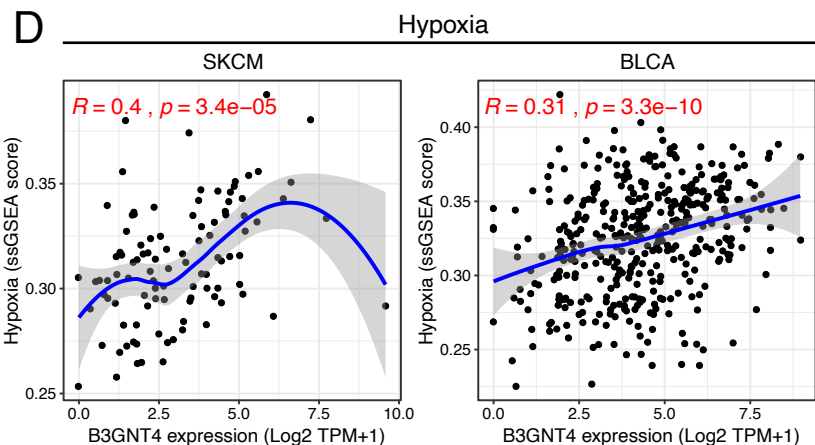

Supplement: Supplementary file 1 — Supporting Information [file CTM2-12-e872-s001.zip › ctm2872-sup-0001-SuppMat/SupplementaryFiles20220516/SFigure/S7.pdf]

A

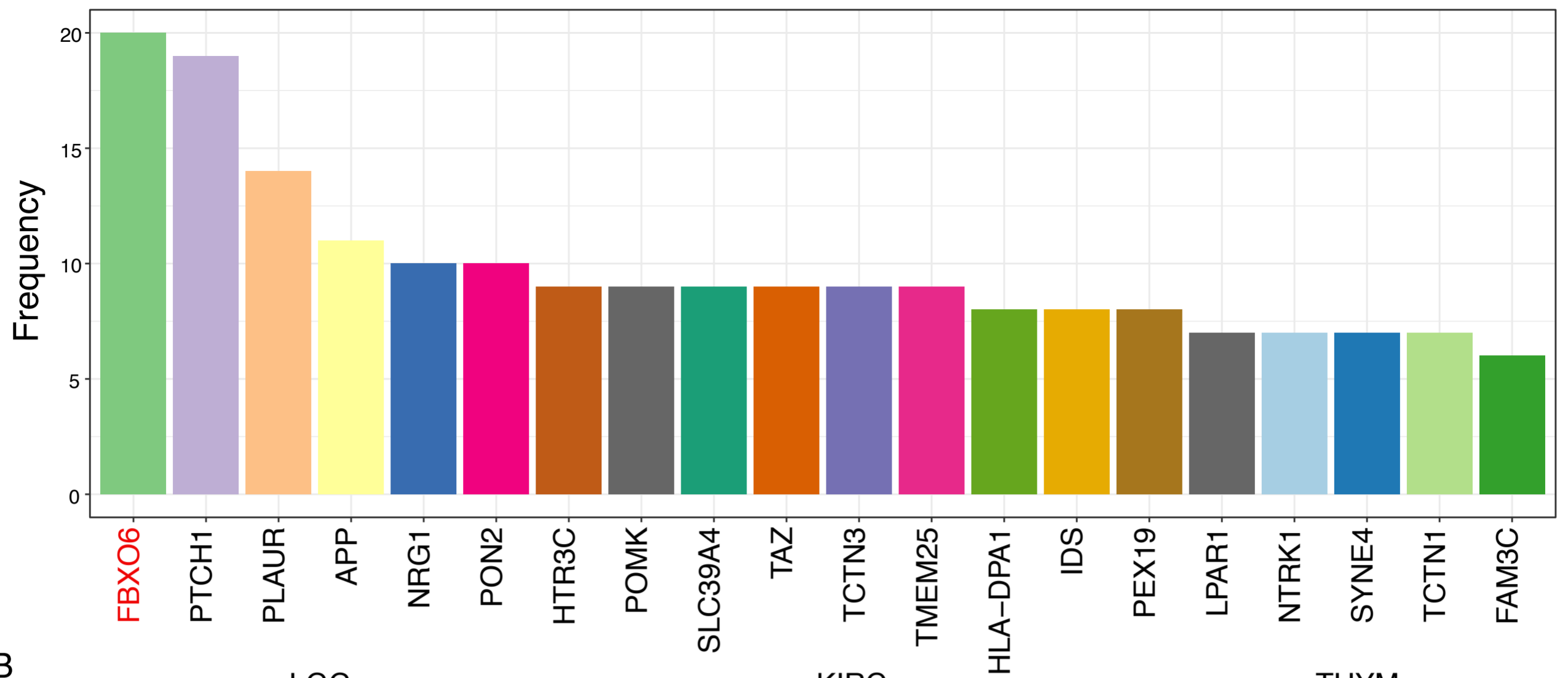

B

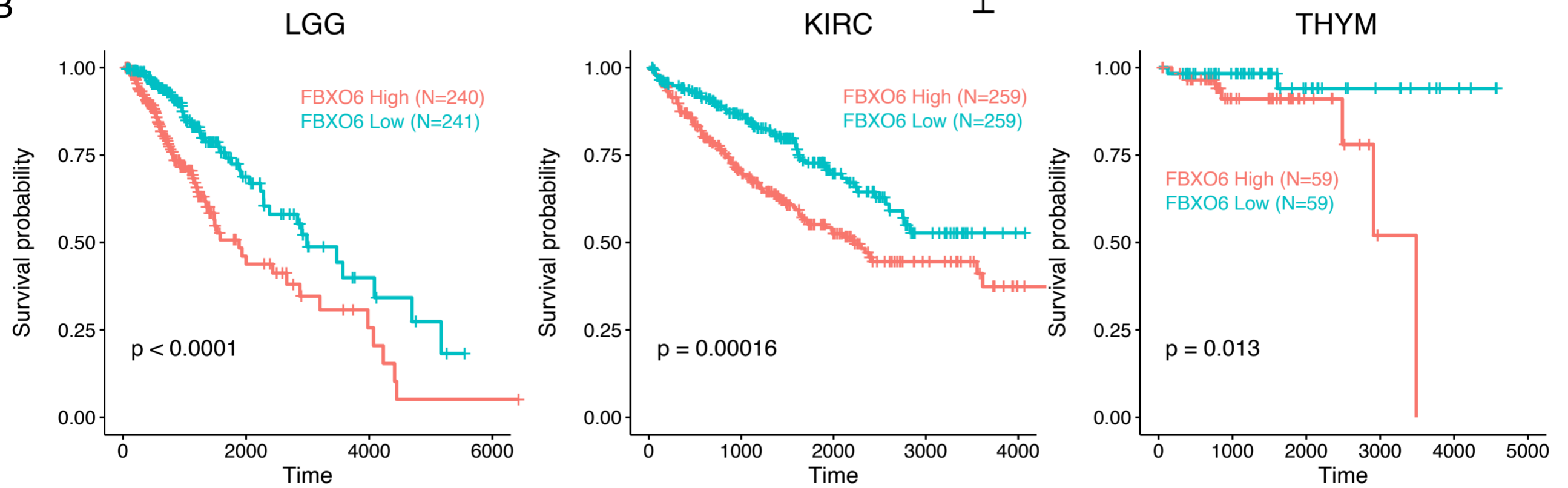

C

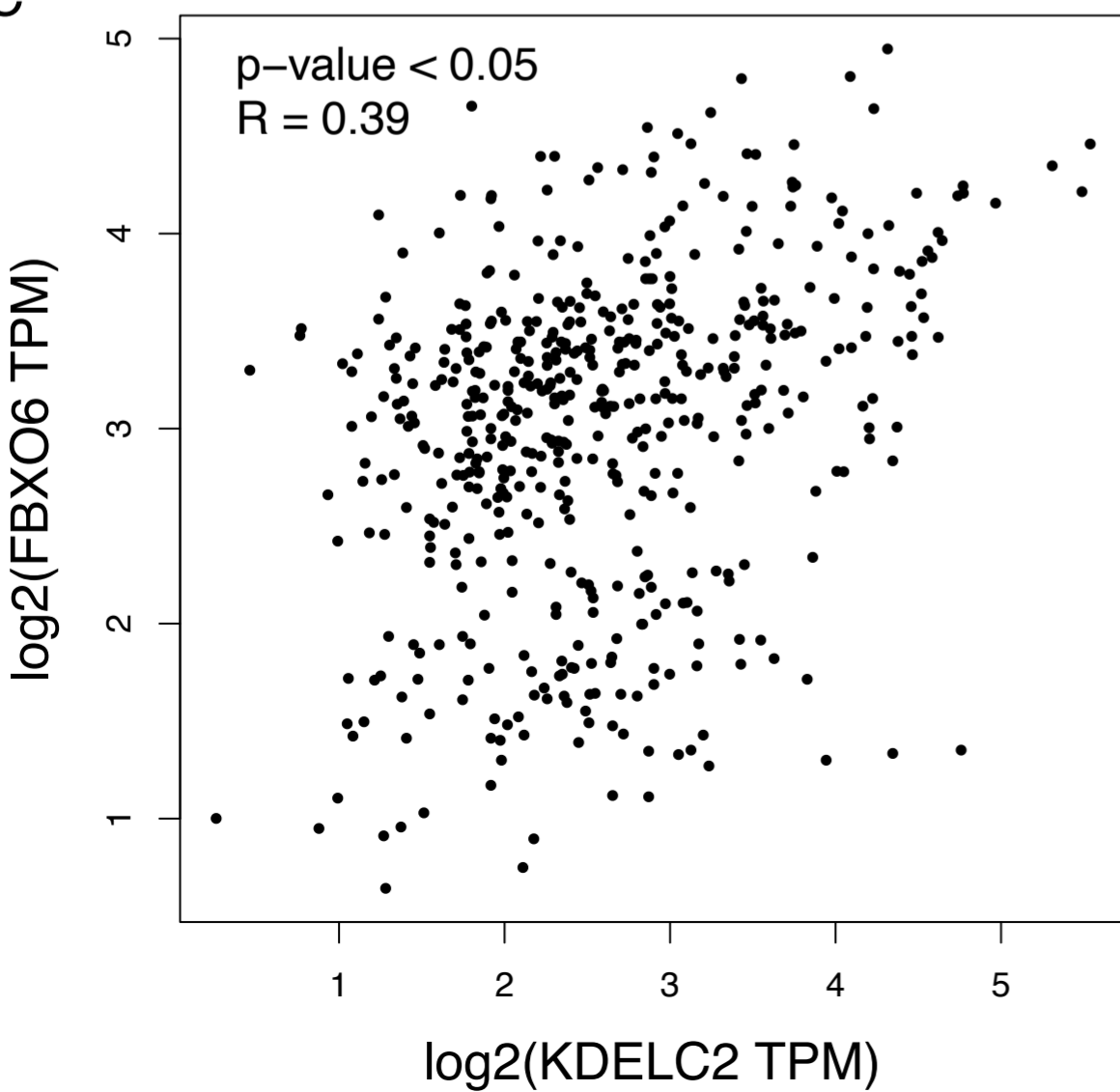

D

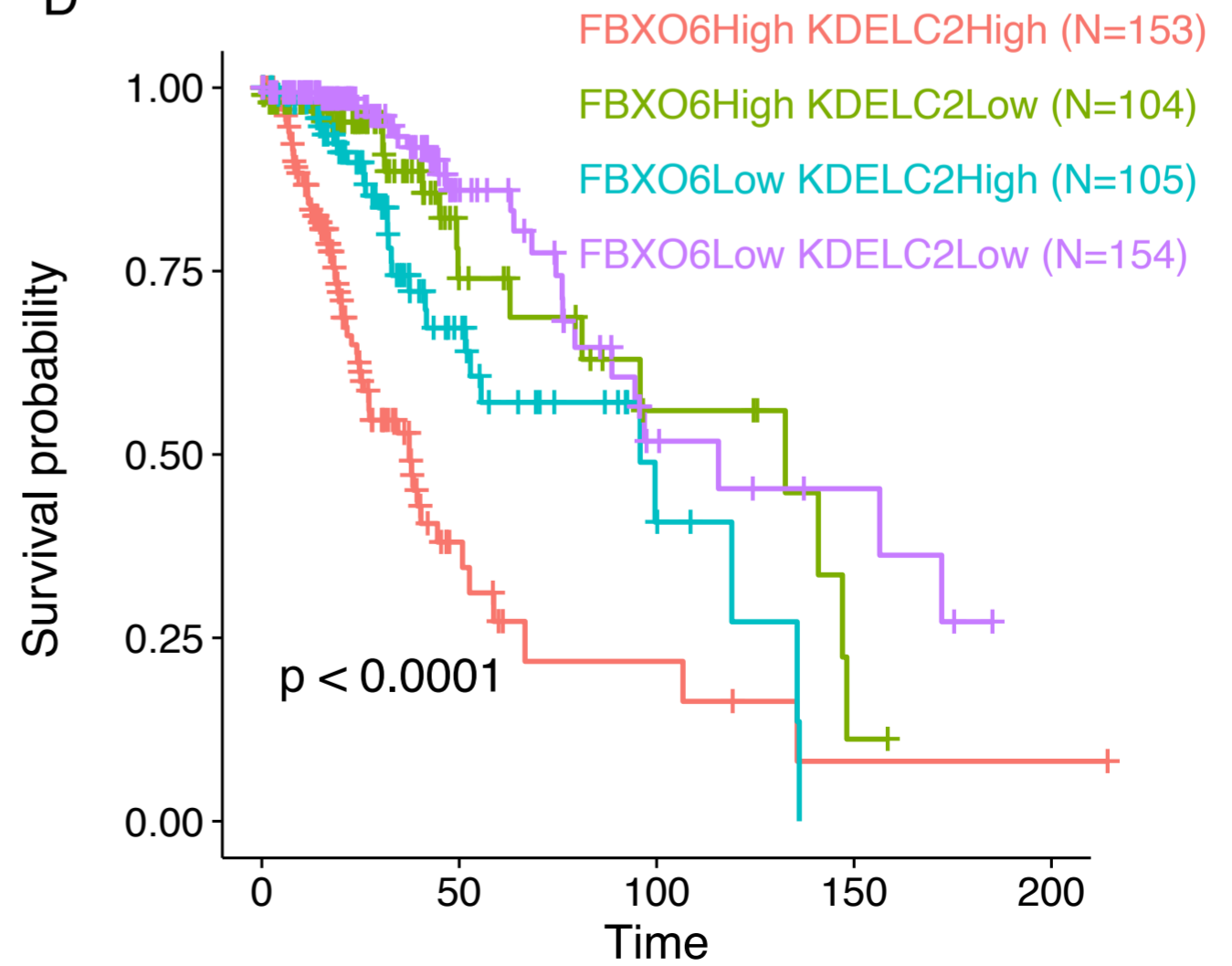

Supplement: Supplementary file 1 — Supporting Information [file CTM2-12-e872-s001.zip › ctm2872-sup-0001-SuppMat/SupplementaryFiles20220516/SFigure/S9.pdf]
